# CD73 on cancer-associated fibroblasts enhanced by the A_2B_-mediated feedforward circuit enforces an immune checkpoint

**DOI:** 10.1038/s41467-019-14060-x

**Published:** 2020-01-24

**Authors:** Miao Yu, Gang Guo, Lei Huang, Libin Deng, Chang-Sheng Chang, Bhagelu R. Achyut, Madison Canning, Ningchun Xu, Ali S. Arbab, Roni J. Bollag, Paulo C. Rodriguez, Andrew L. Mellor, Huidong Shi, David H. Munn, Yan Cui

**Affiliations:** 1 Department of Biochemistry and Molecular Biology, Cancer Immunology, Inflammation & Tolerance Program, Georgia Cancer Center, Augusta University, Augusta, GA 30912 USA; 2 Translational & Clinical Research Institute, Faculty of Medical Sciences, Newcastle University, Newcastle upon Tyne, NE2 4HH UK; 3 Institute of Translational Medicine, Nanchang University, Nanchang, 30000 Jiangxi China; 4 Bioinformatics Shared Resource and Integrated Genomics, Georgia Cancer Center, Augusta University, Augusta, GA 30912 USA; 5 Department of Biochemistry and Molecular Biology, Tumor Signaling & Angiogenesis, Georgia Cancer Center, Augusta University, Augusta, GA 30912 USA; 6 School of Medicine, Augusta University, Augusta, GA 30912 USA; 7 Flow cytometry Core, Georgia Cancer Center, Augusta University, Augusta, GA 30912 USA; 8 Tumor Tissue and Serum Biorepository, Georgia Cancer Center, Augusta University, Augusta, GA 30912 USA; 9 Department of Immunology, H. Lee Moffitt Cancer Center and Research Institute, Tampa, FL 33612 USA; 10 Department of Biochemistry and Molecular Biology, Molecular Biology & Biomarkers, Georgia Cancer Center, Augusta University, Augusta, GA 30912 USA

**Keywords:** Cancer microenvironment, Immunotherapy, Immunosuppression, Cancer microenvironment, Cancer models

## Abstract

CD73, an ecto-5′-nucleotidase (NT5E), serves as an immune checkpoint by generating adenosine (ADO), which suppresses immune activation through the A_2A_ receptor. Elevated CD73 levels in tumor tissues correlate with poor clinical outcomes. However, the crucial source of CD73 activity within the tumor microenvironment remains unspecified. Here, we demonstrate that cancer-associated fibroblasts (CAFs) constitute the prominent CD73^hi^ population in human colorectal cancers (CRCs) and two CD73^−^ murine tumor models, including a modified CRC. Clinically, high CAF abundancy in CRC tissues correlates strongly with elevated CD73 activity and poor prognosis. Mechanistically, CAF-CD73 expression is enhanced via an ADO-A_2B_ receptor-mediated feedforward circuit triggered by tumor cell death, which enforces the CD73-checkpoint. Simultaneous inhibition of A_2A_ and A_2B_ pathways with CD73-neutralization synergistically enhances antitumor immunity in CAF-rich tumors. Therefore, the strategic and effective targeting of both the A_2B_-mediated ADO-CAF-CD73 feedforward circuit and A_2A_-mediated immune suppression is crucial for improving therapeutic outcomes.

## Abstract (web summary)

Our understanding on how CAFs can be activated to support tumour progression is still limited. Here, the authors demonstrate that adenosine produced in the tumour microenvironment can enhance the expression of CD73 in CAFs ultimately driving CD8 T-Cell suppression and tumour growth.

## Introduction

CD73 is an ecto-5′-nucleotidase (NT5E) that generates extracellular adenosine (eADO) through coordination with ecto-nucleoside ATPases, including ectonucleoside triphosphate diphosphohydrolases1 (ENTPD1, NTPDase1, CD39) and NTPDase2 (CD39L1)^1–3^. Extracellular ADO imposes immune inhibition via the adenosinergic A_2A_ receptor^4–6^. Therefore, CD73, as a rate-limiting enzyme of eADO generation, serves as a crucial metabolic and immune checkpoint by diverting the eATP-triggered immune activation signal to eADO-induced immunosuppression. Clinically, elevated CD73 levels in the tumor tissue of several cancer types, including breast, ovarian, and colorectal cancers (CRC), are linked to poor patient survival^7–9^, which underscores the crucial role of CD73 in tumor progression. Currently, CD73 neutralization therapy, either alone or in combination with an A_2A_ antagonist, is being tested in clinical trials. However, the crucial populations targeted by anti-CD73 within the tumor microenvironment (TME) are not clearly defined. Although CD73 expression had been observed on some tumors, such as melanomas and prostate cancers, and tumor-infiltrating leukocytes (TILs), especially regulatory T cells (Tregs)^2,7,10–12^, experimental model systems using *Cd73*^null^ mice clearly suggest a crucial contribution of host CD73 activity in the non-hematopoietic compartment to the observed immunosuppression^12–14^. Aside from endothelial vasculature^12^, CD73 activity in cancer-associated fibroblasts (CAFs), which are the dominant non-hematopoietic stromal cells in many tumors^15–17^, remains largely undefined.

CAFs are known to be crucial for supporting tumor progression, chemoresistance, metastasis, and maintenance of cancer stem cells through the production of growth factors, chemokines, and extracellular matrix (ECM)^15–19^. Recent evidence suggests that CAFs also play active roles in shaping the immune landscape of the TME via ECM-regulated immune cell anchorage and trafficking and via suppression of immune activation^15–17^. Although our understanding of the origin and characteristics of CAFs is still evolving, existing evidence suggests that CAFs are derived from normal fibroblasts (FBs), including tissue resident FBs and bone marrow-derived mesenchymal stem cells (MSCs), which are subsequently modulated by factors and cellular interactions in the TME^20,21^. Interestingly, CD73 has been used as one of the MSC markers^22^, which compelled us to examine CD73 expression on CAFs and whether CAF-CD73 in the TME enforces the immune checkpoint pathways.

Using clinical CRC specimens, publicly accessible transcriptome datasets from human CRC-tissues, CRC-CAFs, and fresh CAFs from mouse tumor models, our comparative investigations demonstrate that CAFs are the prominent CD73^hi^ cells in the TME. Compared with other cellular constituents within the TME, CD73 expression and bioactivity on CAFs are significantly higher and high levels of CD73 in the TME of human CRCs are associated with high CAF abundancy and immunosuppression. Mechanistically, in vivo studies reveal that CAF-CD73 is dynamically regulated by eADO generated in the TME via activation of the A_2B_ receptor. Thus, this CAF-CD73-ADO-A_2B_ feedforward circuit enforces the CD73-checkpoint and works synergistically with the ADO-A_2A_-mediated T cell/immune suppression through two functionally non-redundant processes. Therapeutically, simultaneous implementation of A_2A_ and A_2B_ antagonism, especially in combinations with CD73 neutralization, leads to a significant improvement in tumor control. Using an engineered CD73-MC38 murine CRC model, we further demonstrate that the collaborative effects of andenosinergic antagonism and CD73-neutralization on tumor control are more effective in the CAF-rich TME.

## Results

### CD73^high^ cells in the human CRCs are mostly activated FBs

**Fig. 1:**
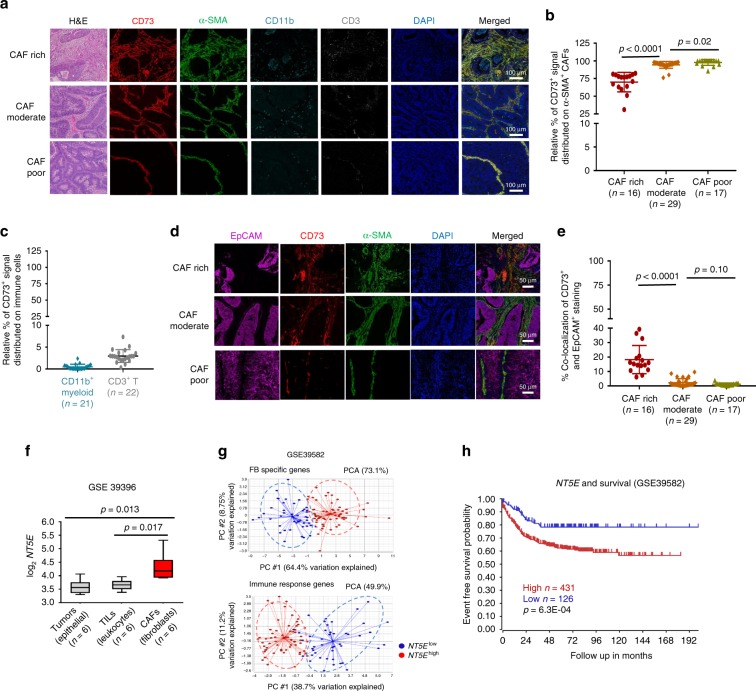
Elevated CD73 levels in human CRCs are associated with CD73^hi^-CAF abundancy and poor clinical outcomes. **a** Histological H&E examination (left) and multiplex immunohistochemistry (IHC) staining of CD73 (red), a-smooth muscle actin (a-SMA, green), CD11b (cyan), and CD3 (gray) were performed with de-identified FFPE CRC-specimen. Nuclei were counterstained with DAPI (blue). Representative images illustrate the level of CD73 expression and its bio-distribution on CAFs (α-SMA^+^), CD11b^+^ myeloid, and CD3^+^ T cells in CAF-rich, moderate, and poor specimens. Scale bars, 100 μm. **b**, **c** Randomly selected and evenly distributed areas (~5 × 10^5^ mm^2^) from each multiplex IHC-stained CRC specimen were analyzed for the percentage of CD73^+^ signal that distributed on α-SMA^+^ **b**, CD11b^+^, and CD3^+^ cells **c** among the total CD73^+^ region in each area, which was set as 100%, was calculated and plotted. **d** Multiplex IHC staining was performed to determine tumor (EpCAM, magenta), CD73 ^+^ (red) cells, and CAFs (α-SMA, green) distribution and their potential co-localization. Scale bars, 50 μm. **e** The percentage of CD73^+^ signal on EpCAM^+^ tumors against total CD73^+^ region was presented. **f** *CD73 (NT5E)* gene expression in paired purified CRC-CAFs (red box), TILs, and tumors from published dataset (GSE39396) was compared. **g** Principle component analysis was performed with a published CRC cohort dataset (GSE39582) of 557 CRC specimens. The *NT5E*^*high*^ (red) and *NT5E*^*low*^ (blue) specimens were first defined by their CD73 expression in the top 20% and bottom 20% level, respectively, and clustered against a group of 18 fibroblast-specific (FB) genes that shows a positive correlation and against a group of 15 pre-defined group immune effector function associated genes as Immune response genes that reveals negative association. **h** The Kaplan–Meier survival curve demonstrates clinical correlation of *NT5E*^*high*^ (red) and *NT5E*^*low*^ (blue) expression in CRC patients (GSE 39582) with event-free survival. Data depict mean ± SEM. Unpaired Student’s *t*-test for **b**, **c**, **e** and paired Student’s *t*-test for **f**. Source data are provided in the Source Data file.

Clinical and molecular investigations demonstrate that CAF abundancy in CRCs is associated with poor prognoses^23,24^. Our examination of 25 formalin-fixed paraffin-embedded (FFPE) or cryopreserved human CRC adenocarcinoma specimens obtained from our tumor bank revealed that more than 50% of the CRCs manifested moderate to high stromal content via H&E staining (Fig. 1a). Multiplex immunohistochemistry (IHC) staining of the FFPE specimens (Fig. 1a) and conventional immunofluorescence (IF) staining of the frozen specimens (Supplementary Fig. 1a) verified the abundant activated FBs, i.e CAFs, using α-smooth muscle actin (α-SMA) or ER-TR7 for reticular fibers^25^ as markers, respectively. Quantitative analysis of CD73 expression and distribution on α-SMA^+^ CAFs, CD11b^+^ myeloid, and CD3^+^ T cells in the IHC-stained specimens via a computer-assisted program indicated that 75–90% of the CD73^+^ signal distributed on α-SMA^+^ CAFs (Fig. 1b), while <5% of the CD73^+^ signal co-localized with CD11b^+^ myeloid and CD3^+^ T cells (Fig. 1c). Similarly, IF-stained frozen specimens confirmed predominate CD73 expression on ER-TR7^+^ CAFs and minimal overlap with CD45^+^ TILs (Supplementary Fig. 1c, d). Moreover, limited CD73 expression was observed in EpCAM^+^ tumor cells in majority of the specimens (Fig. 1d, e), except for a few CAF-rich specimens where CD73^hi^ signal was detected in the lumina (Supplementary Fig. 1e). These histological assessments demonstrate that CD73 protein expression is highly concentrated on fibroblastic stroma or CAFs. Next, we analyzed the publicly accessible human transcriptome dataset of FACSort-purified paired CRC fibroblastic stroma (CAFs), TILs, and epithelial tumor cells (GSE39396), which substantiates the significantly higher *NT5E* mRNA levels in CRC-CAFs than in tumors and TILs (Fig. 1f). Together, these results strongly suggest that CD73^hi^ expression is a unique characteristic of human CRC-CAFs.

Elevated tumor CD73 levels are associated with poor clinical outcomes in several tumor types^7,9,26–28^. Nevertheless, most of the correlative studies were based on CD73 expression in unfractionated tumor tissues. To determine whether a specific algorithm could be formulated to evaluate relative CAF abundancy from the published datasets of total tumor tissues, we defined an 18-gene set of FB-specific genes based on a previous report of highly expressed genes in CRC-CAFs^23^ and those commonly used to define CAFs or FBs^17,20,23^ (Source data for supplementary figures). Likewise, a set of 15 immune response-associated genes was designated from the common genes associated with immune effector, signaling, and function (Source data for supplementary figures). The specificity of these gene sets was validated by at least a two-fold higher expression in purified CRC-CAFs or CRC-TILs, respectively, and distinct clustering of CAFs, tumors, and TILs based on the expression profiles (GSE39396, Supplementary Fig. 1f). Using these two gene-sets, we performed a principal component analysis (PCA) of a CRC transcriptome dataset with 585 patients (GSE39582), and clustered the data at the top 20% of *NT5E* levels (*NT5E*^high^) and bottom 20% of *NT5E* (*NT5E*^low^) groups. As expected, using the FB-gene set as the first principle component (PC #1), *NT5E*^high^ and *NT5E*^low^ groups were segregated into the FB-high and FB-low clusters, respectively (Fig. 1g), confirming a positive correlation between *CD73* expression and CAF abundancy. Likewise, clustering against the immune response gene set positioned the *NT5E*^high^ and *NT5E*^low^ into the immune-response low and high clusters, respectively, supporting a negative correlation (Fig. 1g and Supplementary Fig. 1g). Importantly, high *NT5E* levels in these 557 patients correlated robustly with poor clinical outcomes (Fig. 1h). These results suggest that high levels of *CD73* expression in the human CRC TME correlate positively with CAF abundancy, augmented immunosuppression, and poor prognosis.

### CAFs are the major source of CD73 activity in the EG7 TME

**Fig. 2:**
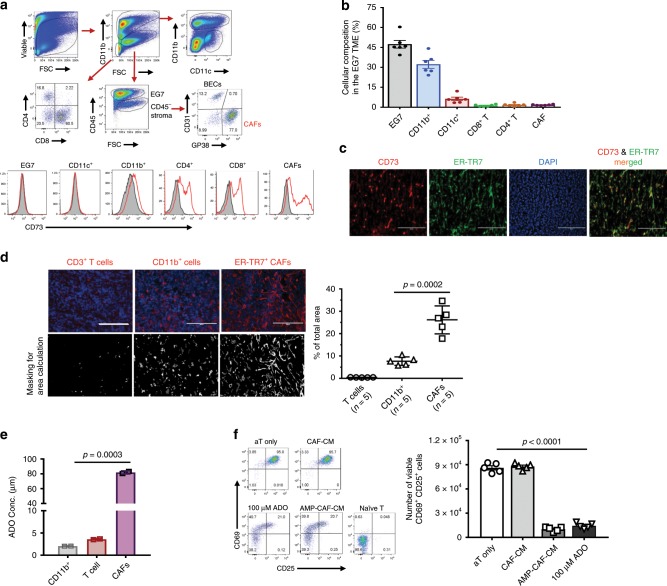
CAFs in the murine TME are CD73^hi^ cells with a superior capacity for ADO generation than TILs. **a** EG7 tumors established s.c. in *C57BL/6* WT mice for 15 days were used for assessment of cellular constituents of the EG7 TME via FACS as CD11b^+^ myeloid cells, CD45^+^CD11b^−^ EG7-tumors (large) and lymphocytes (small cells), and CD45^−^stroma. CD45^−^ cells were further defined as GP38^+^CD31^−^ CAFs and GP38^−^CD31^+^ BECs. CD73 expression in each cellular subset in the TME was examined (red histogram) as compared with the isotype control for each population (gray). **b** Relative percentage of each cellular subset within the TME determined via FACS was summarized. *n* = 6. **c** IF staining was employed to examine CD73^+^ cell (red) bio-distribution in correlation with ER-TR7^+^ (green) fibroblastic stroma and DAPI nuclear counterstain (blue). Co-localization of CD73^high^ and ER-TR7^+^ cells was illustrated via merging the images. **d** The relative area occupied by CD3^+^ T, CD11b^+^ myeloid cells, and ER-TR7^+^ CAFs (red) in the EG7 TME was determined via a computer-assisted software following masking it to white/gray signals and calculated as the percentage of total area. **e** CD11b^+^, CD3^+^ cells, and CAFs were purified from the EG7 TME via FACSort. Their capacity for ADO generation within 2 h in the presence of 200 μM AMP was compared. **f** Purified CD8 T cells were activated by α-CD3/α-CD28 beads in CAF-conditioned medium (CAF-CM), pre-incubated with or without 200 μM AMP. Changes in the cell surface expression of CD69 and CD25 were examined and compared with the 100 μM ADO control via FACS 15 h post-activation. The viable CD69^+^CD25^+^CD8^+^ T cells were calculated and summarized to the right. Error bars depict mean ± SEM. *p* values were determined via two-tail unpaired Student’s *t*-test. All experiments were repeated at least two times. Source data are provided in the Source Data file.

For comparative analysis of the CD73 expression and function among all cellular subsets of the TME, we employed ectopic murine tumor models. Among the MC38-CRC, EG7 T-lymphoma, and B16 melanoma models, CAFs were mostly CD73^hi^ cells compared with TILs and tumors (Fig. 2a, Supplementary Fig. 2a, b). Unlike CD73^−/lo^ human CRC-adenocarcinomas (Fig. 1), MC38 tumors were CD73^+^ with a comparable level of CD73 to that of MC38-CAFs (Supplementary Fig. 2b). Because EG7 tumors were CD73^−^ with abundant CAFs and CAF-CD73 levels were comparable to those seen in human CRCs (Supplementary Fig. 2b–d), we first employed the EG7 model. IF staining confirmed that CD73^hi^ signal distributed throughout the EG7 TME, mostly overlapped with the ER-TR7^+^ CAFs (Fig. 2c). Computer-assisted image analysis revealed that EG7-CAFs occupied up to 30% of the area (Fig. 2b, d) despite only constituting 2–3% of the cellularity determined by flow cytometry (FACS, Fig. 2a, b). In contrast, CD11b^+^ myeloid and CD3^+^ T cells only covered ~10% and 0.5% of the area although they accounted for ~30% and 5% of the cellularity, respectively (Fig. 2b, d). Therefore, similar to human CRC-CAFs, EG7-CAFs exemplify a unique CD73^hi/+^ population that forms an extensive network within the TME.

Using FACSort-purified CAFs (Supplementary Fig. 2e), CD3^+^ T, and CD11b^+^ myeloid cells from the EG7 TME, we compared their CD73 bioactivity as the capacity of ADO generation via an HPLC-based quantification assay (Supplementary Fig. 2f). We added 200 μM AMP to the CAF culture because early studies indicated that ADO concentrations in the TME could reach 50–100 μM^29,30^. EG7-CAFs generated close to 100 μM ADO within 4 h (Fig. 2e), which could be inhibited by a CD73 inhibitor, adenosine 5′-(α,β-methylene) diphosphate (APCP) (Supplementary Fig. 2g). In contrast, the same number of purified CD11b^+^ or CD3^+^ cells from the same TME generated < 5 μM ADO (Fig. 2e). Moreover, the CAF culture medium in the presence of AMP (AMP-CAF-CM), but not in the absence of AMP (CAF-CM), markedly inhibited T cell activation and proliferation similar to that induced by 100 μM ADO (Fig. 2f, Supplementary Fig. 2h). Together, our results confirm that CD73 on EG7-CAFs plays a dominant role in rapid ADO generation and immunosuppression.

### EG7 and human CRC CAFs share similar transcriptome profiles

**Fig. 3:**
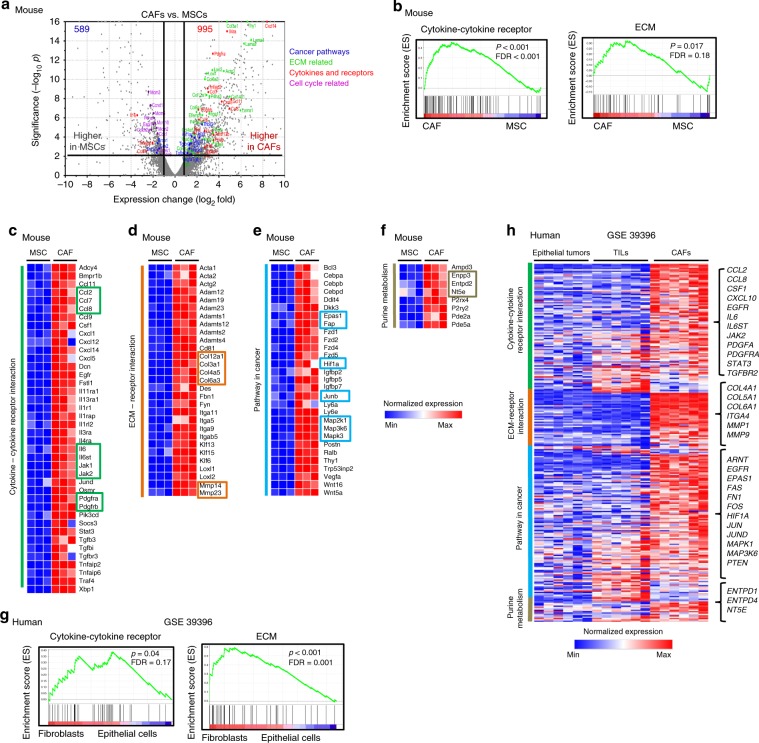
EG7 and human CRC CAFs share similar transcriptome profiles. **a** Comparative expression profile of CAFs and MSCs was analyzed via RNA sequencing. The volcano plot illustrates genes that were significantly upregulated (995, red) and downregulated (589, blue) adjusted for fold change > 2 and *p* < 0.01 with representative genes associated with specific pathways. **b** Gene sets specifically enriched among the differentially expressed genes in CAFs were analyzed via gene set enrichment analysis (GSEA). **c**–**f** The 417 genes categorized in “response to stimulus” were further analyzed for enrichment in KEGG pathways. Representative heatmaps of RNA sequencing data with genes significantly up-regulated in CAFs according to specific pathways were shown from three biological repeats, each extracted from at least five pooled tumor-bearing mice. **g** Gene set enrichment analysis (GSEA) of differentially expressed genes in human CRC-CAFs compared with paired tumor cells (GSE39396) identified highly enriched genes in pathways related to cytokine–cytokine interaction and ECM receptor interaction. **h** The gene expression profile heatmap shows differentially expressed genes in paired human CRC tumors, TILs, and CAFs (GSE39396) associated with pathways highly enriched in CRC-CAFs. The list on the right showed a selective representative shortlist of significantly up-regulated genes in CAFs, most of which are similar to those identified in EG7-CAFs. Source data are provided in the Source Data file.

The dynamic and mutualistic interactions between stroma and tumors in the TME shape the immune landscape and modulate CAF function and phenotype^17^. Despite the differences in tumor type and species between EG7-CAFs and human CRC-CAFs, we postulated that they share many similar cellular and molecular characteristics besides the CD73^hi^ phenotype and, thus, compared their transcriptome profiles. First, comparative high throughput RNA-sequencing (seq) analysis of purified EG7-CAFs and the early passage murine MSCs, which are thought to be precursors for some CAFs^16,20,31^ and used as the control for normal mesenchymal cells. With a two-fold difference and a significance of *p* < 0.01 from 11,839 genes, 1584 differentially expressed genes (DEGs) between CAFs and MSCs were identified (Fig. 3a). These DEGs were pertinent to various pathways, including response to stimulus, metabolism, cancer pathways, ECM, and cell cycle regulation (Fig. 3a and Supplementary Fig. 3a). Gene set enrichment analysis (GSEA) elucidated that genes associated with cytokine–cytokine receptor signaling and ECM were highly enriched in EG7-CAFs (Fig. 3b), consistent with their immune regulatory function and involvement in tissue remodeling. Further analysis of the 417 genes upregulated as response to stimulus via KEGG pathway grouped them into pathways in cancer, ECM, cytokine–cytokine receptor interaction, purine metabolism, etc. (Supplementary Fig. 3b). As expected, cytokine/chemokine and signaling molecules, including various interleukins (IL), IL-receptors, downstream transcription factors, *Ccl*s, *Cxcl*s, *Tgf*s, *Jak*s, *Stat*s, and *Socs3*, were highly elevated in CAFs (Fig. 3c). Likewise, genes associated with ECM, hypoxia, and purine metabolism, such as *Col*s, *Mmp*s, *Hif1α*, *Epas1* (*Hif2α*), *Nt5e* (*Cd73*), and *Entpd2* (*Cd39l1*), were highly elevated in CAFs compared with MSCs (Fig. 3d–f). Notably, common markers used for defining CAFs, including *Pdgfra*, *Pdgfrb* (Fig. 3c), *Acta2*, *Des* (Fig. 3d), *Fap, Postn*, and *Thy1* (Fig. 3e), were all expressed at higher levels in CAFs than in MSCs. Collectively, our results clearly support the notion that CAFs are transcriptionally different from MSCs in various pathways and biological processes associated with immune modulation, inflammation, hypoxia, and activation of oncogenic pathways.

We next analyzed GSE39396 transcriptome dataset of the paired CRC-CAFs, TILs, and tumors. Considering higher individual variations among clinical specimens, we used a 1.5-fold difference with a significance of *p* < 0.01 and identified 2927 DEGs that expressed at higher levels in CRC-CAFs than in tumors and TILs. Similar to EG7-CAFs, GSEA demonstrated highly enriched gene expression associated with cytokine–cytokine receptors and ECM in CRC-CAFs (Fig. 3g). KEGG analysis also categorized these DEGs into pathways similar to those of EG7-CAFs (Supplementary Fig. 3c). Remarkably, many of the DEGs identified in human CRC-CAFs (Fig. 3h) mirror those in EG7-CAFs (Fig. 3c–f). Together, this comparative transcriptome profiling confirms common characteristics at the molecular level shared between murine and human CAFs from different TME, supporting the clinical relevance of our EG7-CAF as a model system for mechanistic investigation of molecular pathways and therapeutic interventions targeting CAF-CD73-dependent immune suppression in the TME.

### CD73 on non-hematopoietic cells promotes tumor progression

**Fig. 4:**
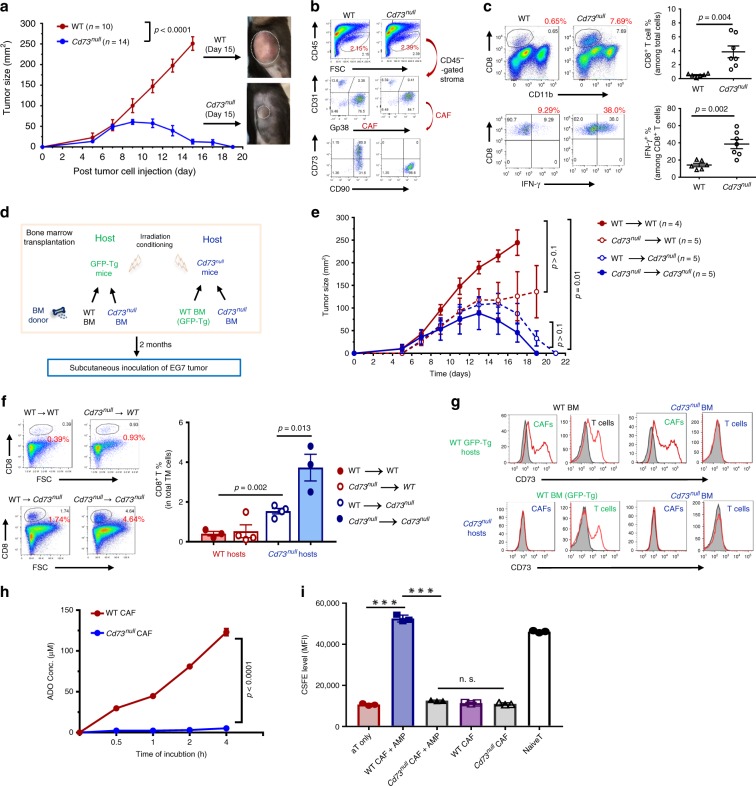
*Cd73* inactivation in the non-hematopoietic compartment hinders tumor progression causing tumor regression. **a** *C57BL/6* WT and *Cd73*^*null*^ mice received 1 × 10^6^ EG7 tumors s.c. were monitored for tumor progression every other day. Representative images illustrate the tumor size in WT and *Cd73*^*null*^ mice at 15 days post-inoculation. **b** At 9 days post-EG7 inoculation, tumors were collected from WT and *Cd73*^*null*^ mice for analysis of EG7-CAF percentage and phenotype. **c** The percentage of tumor infiltrating CD8^+^ T cells and IFN-γ^+^ effectors in the EG7 TME of WT and *Cd73*^*null*^ mice were analyzed, summarized, and shown to the right. **d** Schematic diagram shows reciprocal BMT strategy between WT *C57BL/6* (GFP-Tg) and *Cd73*^*null*^ mice. **e** Two months post-BMT, EG7 tumors were established s.c. in these BMT mice and tumor progression was examined every other day. **f** Representative FACS plots (left) and summary (right) of tumor-infiltrating CD8^+^ T cells in the BMT mice are presented. **g** The representative histograms show CD73 expression (red line) compared with isotype control (gray) in CAFs and T cells of the BMT mice. **h** EG7-CAFs from WT and *Cd73*^*null*^ mice were purified via FACSort. Their capacity and kinetics for ADO conversion from 200 μM AMP was determined. **i** The capacity of WT and *Cd73*^*null*^ CAF-conditioned medium in the absence (CAF-CM) or presence of 200 μM AMP (AMP-CAF-CM) for inhibiting T cell proliferation as CFSE dilution was examined. Data depict mean ± SEM. Student’s *t*-test. *n* = 5. Source data are provided in the Source Data file.

We first evaluated the impacts of host CD73 activity on tumor growth by comparing EG7 progression in *Cd73*^null^ and WT mice. As reported previously^12,13^, despite their similar tumor establishment during the first week of tumor inoculation (Fig. 4a), EG7 progression in *Cd73*^null^ mice was hindered during the second week, when tumors reached ~70–100 mm^2^, followed by regression and elimination (Fig. 4a). In contrast, EG7 in WT mice progressed rapidly and required euthanasia by day 15 (Fig. 4a). Examination of the EG7 tumors established in *Cd73*^null^ and WT mice for 9 days with similar size demonstrated their comparable CAF abundancy, distribution, and phenotype, except for CD73 expression (Fig. 4b and Supplementary Fig. 4a). Consistent with the observed EG7 regression, CD8^+^ cells and IFN-γ producing effectors, as well as the CD11c^+^ myeloid/dendritic cells, increased significantly in the TME of *Cd73*^null^ mice compared with those of the WT counterparts (Fig. 4c, Supplementary Fig. 4b).

To verify the contribution of CAF-CD73, we performed reciprocal bone marrow transplantations (BMTs) by transferring BM from WT or *Cd73*^null^ mice to lethally irradiated GFP-transgenic mice (GFP-Tg) and BM from GFP-Tg or *Cd73*^null^ mice to irradiated *Cd73*^null^ mice (Fig. 4d) followed by EG7 inoculation 2 months later. As expected, EG7 progressed rapidly in WT mice that received WT BM (Fig. 4e, WT → WT), but slightly delayed in WT mice that received *Cd73*^null^ BM (Fig. 4e, *Cd73*^null^ → WT). Strikingly, *Cd73*^null^ mice that received either WT or *Cd73*^null^ BM (Fig. 4e, WT → *Cd73*^null^ and *Cd73*^null^ → *Cd73*^null^) experienced complete tumor regression resembling un-manipulated *Cd73*^null^ mice (Fig. 4a). Similarly, the tumor regression in *Cd73*^null^ BMT mice was associated with a marked increase in CD8^+^ cells and IFN-γ-producing effectors (Fig. 4f). FACS analysis confirmed that CD73 expression on CAFs matched the recipient genotype, while CD73 expression on T cells agreed with their donor genotype (Fig. 4g). Functionally, *Cd73*^null^ CAFs were unable to generate ADO, confirming inactivated enzymatic activity (Fig. 4h) and inability to inhibit T cell activation and proliferation (Fig. 4i and Supplementary Fig. 4c). Therefore, *Cd73* inactivation in the non-hematopoietic compartment in the EG7 TME, predominately CAFs, leads to enhanced antitumor immunity and consequential tumor elimination.

### CAF-CD73 is dynamically regulated via the ADO-A_2B_ pathway

**Fig. 5:**
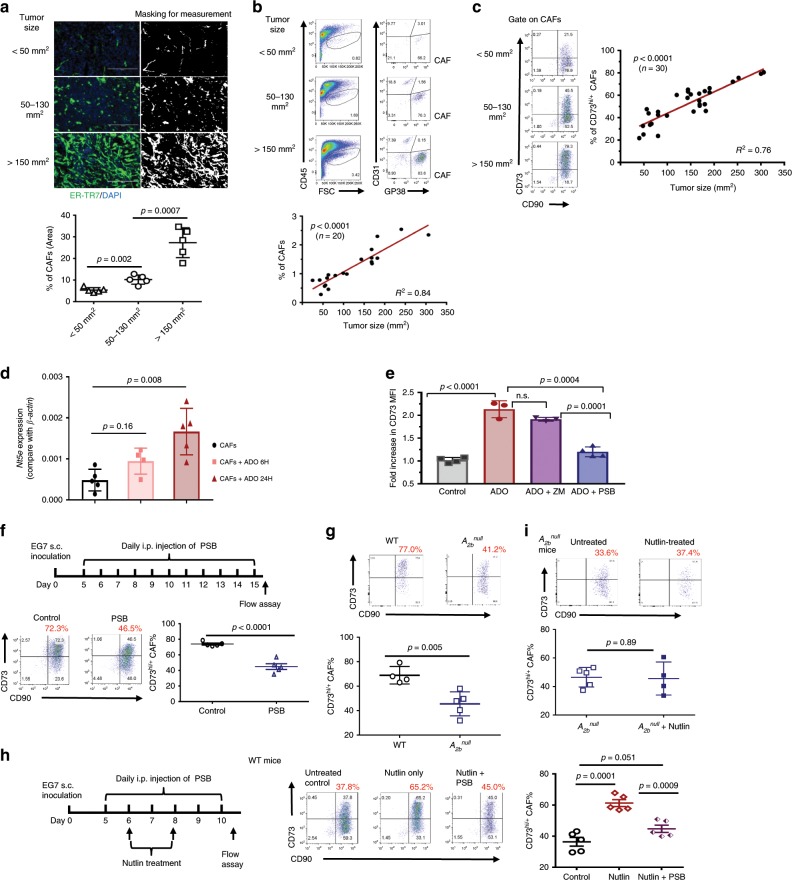
CAF-CD73 expression is dynamically upregulated via the ADO-A_2B_ pathway. **a** The relative abundancy and distribution of CAFs in EG7 tumors of various sizes were evaluated via ER-TR7 (green) staining using a computer-assisted software, MetaMorph. Data depict mean ± SEM. **b**, **c** The CAF abundancy and CAF-CD73 levels in EG7 of various sizes were analyzed via FACS. **d** EG7-CAFs were treated with 100 μM ADO for 6 and 24 h and their *Nt5e* (*Cd73)* expression was determined via quantitative real-time RT-PCR. **e** EG7-CAFs were treated with 100 μM ADO in the absence or presence of A_2A_ or A_2B_ antagonists, ZM241385 or PSB1115, respectively. Relative CD73 expression on CAFs was determined via FACS. **f** EG7 tumor-bearing WT mice were treated with daily i.p. injection of 1 mg kg^−1^ body weight of PSB1115 or vehicle (DMSO) as a control. CD73 expression on CAFs were examined via FACS. **g** WT and *A*_*2b*_^*null*^ mice were inoculated with EG7 tumors and their CAF-CD73 expression from tumors of similar sizes was compared. **h** EG7 tumor-bearing WT mice were treated with daily i.p. injection of 1 mg kg^−1^ body weight of PSB1115 or vehicle starting from 5-day post-tumor inoculation followed with i.t. injections of 100 μl of 20 μM nutlin at days 6 and 8. **i** EG7 tumor-bearing *A*_*2b*_^*null*^ mice were treated with i.t. injections of 100 μl of 20 μM nutlin at days 6 and 8 post-tumor establishment. CD73 expression on CAFs from WT **h** and *A*_*2b*_^*null*^ **i** mice were analyzed via FACS and compared to those without nultin-treatment (*n* = 5). Error bars depict mean ± SEM. *p* values were determined via two-tail unpaired Student’s *t*-test **a**, **d**–**i**. Linear regression analysis was performed to determine the correlation between the tumor size and CAF% or CD73^+^CAF% in **b**, **c**, respectively. All experiments were repeated at least two times. Source data are provided in the Source Data file.

During tumor progression, cancers and other cellular constituents in the TME communicate dynamically and multidimensionally to shape the immunosuppressive landscape. However, it is largely unexplored when/how CAF-network is established and how CD73 activity is regulated. We analyzed EG7 tumors of various sizes and found that CAFs were sparse in small tumors (<50 mm^2^), covering < 5% of the area and comprising ~0.8% cellularity (Fig. 5a, b). As tumors progressed, CAFs transiently expanded to ~10% of the area and ~1.7% cellularity (medium-size, 50–130 mm^2^), and eventually formed a well-connected dense network that occupied ~30% of the area and ~3.4% cellularity in large tumors (>150 mm^2^) (Fig. 5a, b). Notably, CAF-CD73 levels increased rapidly during tumor progression from about 20% CAFs being CD73^+^ in small tumors to up to 80% CD73^+^ in large tumors (Fig. 5c and Supplementary Fig. 5a). Interestingly, a progressive increase in tumor hypoxia and necrosis/apoptosis during EG7 progression was observed (Supplementary Fig. 5b), agreeing with previous documentations of tumor growth-associated hypoxia, localized cell death, and activation of multiple pathways^32,33^.

To delineate the molecular mechanism by which CAF-CD73 is upregulated, we first cultured EG7-CAFs and MSCs in hypoxia chambers (1% O_2_) and found that neither *Nt5e* mRNA nor surface CD73 expression was elevated despite their marked upregulation of hypoxia-associated genes, *hypoxia-inducible factor 1 alpha* (*Hif1α)* and *Vegfa* (Supplementary Fig. 5c, d). Thus, unlike the early report of HIF-1α-dependent CD73 upregulation in intestinal epithelium^34^, CD73 expression in fibroblastic stroma is regulated via other mechanisms. Because tumor hypoxia-induced cell death leads to elevated ADO in the TME^29,30,35^, we then cultured CAFs in the presence of ADO, which markedly elevated *Nt5e* expression within 24 h (Fig. 5d). Adenosinergic receptors of various subtypes are constitutively, but differentially expressed in different tissues^3,36,37^. Our RNA-seq results suggested that *Adora2a* (*A*_*2a*_) and *Adora2b* (*A*_*2b*_) are the major subtypes expressed in the fibroblastic stroma. Comparative analysis of their expression in CAFs, MSCs, and T cells via real-time RT-PCR revealed that T cells predominately expressed *A*_*2a*_ with a barely detectable *A*_*2b*_, whereas CAFs and MSCs preferentially expressed 10–50 fold higher *A*_*2b*_ than *A*_*2a*_ (Supplementary Fig. 5e). Treatment of EG7-CAFs with ADO in the presence of either A_2A_ or A_2B_ antagonist, ZM241385 (ZM) or PSB1115 (PSB), respectively, demonstrated that A_2B_ but not A_2A_ antagonist prevented ADO-induced CD73 upregulation (Fig. 5e). Furthermore, in vivo treatment of EG7-bearing WT mice with daily PSB1115 injection markedly suppressed CD73^+^ CAFs by about 50%, without affecting CAF abundancy, compared to those in vehicle-treated control (Fig. 5f, Supplementary Fig. 5f). Notably, the effects of PSB1115 on CAF-CD73 expression were cell-type specific because CD73 levels in CD4 T cells in the same TME were unaffected (Supplementary Fig. 5g). Consistent with these observation, CD73 levels on EG7-CAFs from *A*_*2b*_^null^ mice were significantly lower than those from their WT counterparts of comparable tumor size (Fig. 5g).

Therapy-induced cell death and tissue damage cause substantial elevation of eADO level associated with massive ATP release and conversion^38,39^, which we speculated to account for one of the mechanisms by which chemotherapy and radiotherapy activate CAFs via activating the ADO-CD73 axis to enhance their pro-tumor and immunosuppressive function^40–42^. Analysis of a published human CRC dataset (GSE 15781) consisting of patients with or without radiotherapy prior to surgery indicated that *NT5E* levels in the TME of patients receiving radiotherapy were significantly higher than those without radiation (Supplementary Fig. 5h). Previously, we showed that intratumoral (i.t.) injection of a p53 activator nutlin-3a (nutlin) to EG7 tumors induced localized cell death within the TME^43^. Here, we demonstrated that nutlin treatment robustly augmented CAF-CD73 upregulation, which could be prevented by PSB treatment during the period of nutlin injections (Fig. 5h). Likewise, in *A*_*2b*_^null^ mice, this nultin-induced CD73 upregulation on EG7-CAFs was alleviated (Fig. 5i), which agreed with the observed failure of ADO to induce CD73 upregulation on *A*_*2b*_^null^ CAFs in the presence of ADO (supplementary Fig. 5i). Altogether, these results suggest that therapy-associated ADO elevation is a direct mediator of CAF-CD73 upregulation via activation of A_2B,_ which initiates a feedforward circuit to amplify the CD73-ADO axis in the TME. A_2B_ antagonist PSB1115 or *A*_*2b*_ inactivation alleviates ADO-mediated CAF-CD73 upregulation, which potentially unleashes the CAF-CD73 checkpoint and enhances antitumor immunity.

### Anti-CD73 with A_2A_ and A_2B_ antagonists inhibits EG7 growth

**Fig. 6:**
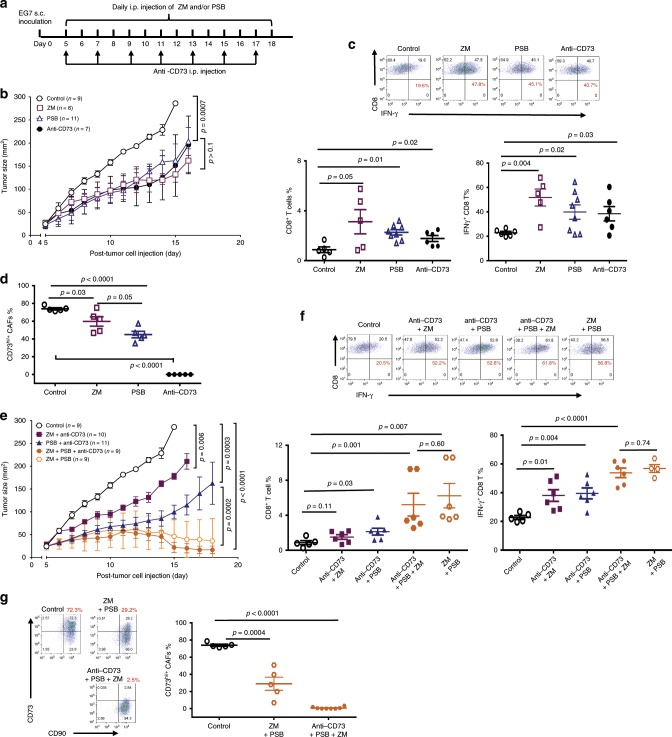
CD73 neutralization in combination with A_2A_ and A_2B_ antagonists leads to significant EG7 tumor regression. **a** Schematic illustration of timeline and treatments of EG7-bearing WT mice with daily i.p. injection of 1 mg kg^−1^ body weight of A_2A_ antagonist ZM241385 or A_2B_ antagonist PSB1115 with or without anti-CD73 treatment. **b** Tumor progression was examined every day. **c** The percentage of TIL-CD8 T cells in the EG7 TME and IFN-g-producing CTLs were determined via FACS and summarized. **d** The percentage of CD73^hi/+^ CAFs in the TME following different treatments were analyzed via FACS. **e** EG7-bearing mice were treated with daily i.p. injection of combined ZM and PSB with or without anti-CD73. Tumor progression was monitored every day. **f** The percentage of TIL-CD8 T cells and IFN-g-producing CTLs in the EG7 TME were analyzed via FACS. **g** The percentage of CD73^hi/+^ CAFs in the TME of various treated groups were analyzed via FACS. **b**, **e** *p* values were determined via multiple *t*-tests where the tumor sizes at each time point were compared. **c**, **d**, **f**, **g** Error bars depict mean ± SEM. *p* values were determined via two-tailed unpaired Student’s *t*-test. All experiments were repeated at least two times. Source data are provided in the Source Data file.

Currently, anti-CD73 blockade, either as a monotherapy or combination with A_2A_ antagonist^44^, is being tested in clinical trials^3^. Based on our observed preferential A_2B_ expression in CAFs and the A_2B_-dependent CD73 amplification circuit, we hypothesized that incorporating the A_2B_ antagonist therapeutically further inhibits the CAF-CD73 immune checkpoint. In EG7-bearing mice, A_2A_ antagonist ZM241385, A_2B_ antagonist PSB1115, or CD73-neutralization individually resulted in a similar modest but statistically significant suppression of EG7 tumor progression (Fig. 6a, b) with marked increases in total TIL-CD8 cells and IFN-γ-producing CTLs compared to those in iso-type control-treated mice (Fig. 6c). Notably, ZM243185 induced the most profound increases in immune effectors (Fig. 6c), whereas PSB1115 treatment markedly inhibited CD73 upregulation in CAFs (Fig. 6d), and CD73-neutralization completely masked CD73 staining on both CAFs and CD4 cells without affecting CAF abundancy (Supplementary Fig. 6a, b). Combinatorial of anti-CD73 together with PSB1115 further improved tumor control and inclusion of A_2A_ and A_2B_ antagonists with CD73-neutralization markedly restrained tumor progression leading to complete tumor elimination in some mice (Fig. 6e). Interestingly, combination of ZM243185 and PSB1115 without anti-CD73 significantly improved tumor control during early phase of the treatment similar to that seen in the three-regimen combination, but was less effective in the later phase of the treatment (Fig. 6e). Consistent with the effective tumor control, the effector frequencies were highest in the ZM243185 + PSB1115 and three-regimen combination groups with ~3-fold increase in TIL-CD8 and CTLs (Fig. 6f). Furthermore, the three-regimen combination imposed a better suppression of CAF expansion and CD73 expression than the ZM243185 and PSB1115 combination (Fig. 6g and Supplementary Fig. 6c), agreeing with the better long-term tumor control in the three-regimen combination group (Fig. 6e). Together, these results demonstrate that blocking the non-redundant functions of A_2A,_ A_2B,_ and CD73 in the TME of CD73^−^ tumors are crucial for maximal reversal of the immune suppression by alleviating the A_2B_-CD73 feedforward circuit in CAFs that enforces the CD73-ADO immune checkpoint.

### A_2B_ blockade in the CAF-poor MC38 fails to suppress CAF-CD73

**Fig. 7:**
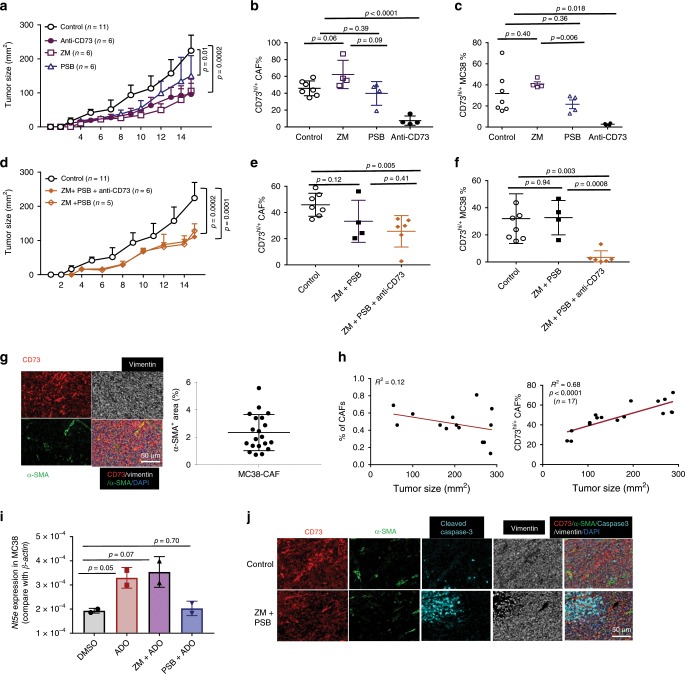
Adenosinergic antagonism is ineffective in modulating CAF-CD73 expression in the CAF-poor MC38 tumors. **a** MC38 tumor-bearing mice were treated with daily i.p. injection of A_2A_ antagonist ZM241385 or A_2B_ antagonist PSB1115 or anti-CD73 every other day. Tumor progression was examined every day. **b**, **c** The percentage of CD73^hi/+^ CAFs (**b**) and MC38 tumors (**c**) in the TME following different treatments were analyzed via FACS. **d** MC38 tumor progression in WT mice receiving combination treatment of ZM243185 and PSB1115 in the absence or presence of anti-CD73 was examined every day. **e**, **f** The percentage of CD73^hi/+^ CAFs (**e**) and MC38 tumors (**f**) in the TME following different treatments were analyzed via FACS. **g** Representative images of IHC staining of CD73 (red), α-SMA (green), and vimentin (white) revealed their levels and distribution within the MC38 TME. The percentage of a-SMA^+^ signal covering the entire TME was determined via the Inform software and presented to the left. **h** MC38 tumor established in WT mice were collected at different sizes for FACS analysis. The relative CAF abundancy and CD73^hi/+^ CAFs within the MC38 TME in association with tumor size were evaluated via linear regression. **i** MC38 tumors were cultured with 100 μM ADO in the absence of presence of 10 mM ZM241385 or PSB115 for 48 h. Their *Cd73* mRNA levels relative to that of *b-actin* was analyzed via real-time RT-PCR. **j** Representative images of IHC staining of CD73 (red), α-SMA (green), apoptotic cells (cleaved caspase-3^+^, cyan) and vimentin (white) levels and their distribution in the MC38 TME treated with or without ZM241385 and PSB115. **a**, **d** *p* values were determined via multiple *t*-tests where the tumor sizes at each time point were compared. **b**, **c**, **e**, **f**, **g**, **i** Error bars depict mean ± SEM. *p* values were determined via two-tailed unpaired Student’s *t*-test. **g**, **h** Scale bars, 50 μm. All experiments were repeated at least two times. Source data are provided in the Source Data file.

We next examined whether these mono- or combinatorial regimens targeting the CD73 and adenosinergic pathways were effective for treating MC38-CRC, which expresses high levels of CD73 with low CAF abundancy (Supplementary Fig. 2). CD73-neutralization, ZM241385, or PSB1115 alone caused a similar and significant delay in MC38 progression (Fig. 7a). Notably, neither ZM241385 nor PSB1115 suppressed CAF-CD73 levels on MC38-CAFs despite a marked suppression of CD73 on MC38 by PSB1115 (Fig. 7b, c). CD73-neutralization alone effectively blocked CD73 detection on both MC38-CAFs and tumors (Fig. 7b, c). Surprisingly, combination of ZM241385 and PSB1115 either in the absence or presence of anti-CD73 did not impose synergistic or additive effects on controlling MC38 progression beyond those observed in monotherapies (Fig. 7d), neither did they effectively suppress CD73 upregulation on MC38-CAFs (Fig. 7e) nor unaltered MC38-CAF abundancy (Supplementary Fig. 7a). Interestingly, CD73-neutralization in ZM241385 and PSB1115-treated mice inhibited CD73 detection on MC38 tumors (Fig. 7f), implying that the accessibility and effects of these therapeutic agents to MC38-CAFs are potentially limited and that the observed control of MC38 progression is likely contributed directly by the agent-mediated tumor killing (Supplementary Fig. 7b) and/or modification of TILs, instead of the CAF-CD73 modification demonstrated in the EG7 TME.

Histologically, MC38 exhibited spindle-shaped mesenchymal-like phenotype (Supplementary Fig. 7c). Transcriptionally, MC38 expressed high levels of FB/mesenchymal-associated molecules, suggesting an epithelial to mesenchymal transition (EMT) phenotype (Supplementary Fig. 7d), which differed markedly from that of typical human CRC-adenocarcinomas (CRC-Tu, Supplementary Fig. 7e). Comparative analysis of FACSort-purified MC38-CAFs (Supplementary Fig. 7f) revealed that they expressed higher levels of *Cd73* mRNA than that of MC38 tumors and EG7-CAFs (Supplementary Fig. 7g). Furthermore, despite a moderate expression in MC38-tumors, *α-Sma* mRNA in MC38-CAFs was a 1000-fold higher (Supplementary Fig. 7h), which allowed for the α-SMA as a marker to distinguish MC38-CAFs from MC38E (Fig. 7g). Strikingly, IHC analysis revealed that MC38-CAFs were scattered sparsely, only covering ~2% of the TME (Fig. 7g) unlike the abundant/sophisticated CAF-networks in the EG7-TME and those in clinical CRC specimens (Fig. 1a). Furthermore, the frequency of MC38-CAFs remained unchanged although the relative percentage of CD73^+/hi^ CAFs elevated gradually during tumor progression (Fig. 7h). In vitro and in vivo studies revealed that MC38 was more susceptible than MSCs to ZM241385-induced cell death (Supplementary Fig. 7i), which likely triggered the ADO-dependent CD73 upregulation in the remaining MC38 cells and adjacent MC38-CAFs (Fig. 7i, j). Consistent with their mesenchymal phenotype, PSB1115 suppressed ADO-induced CD73 upregulation in MC38 cells (Fig. 7i) with modest toxicity (Supplementary Fig. 7j). Together, these results support our hypothesis that the therapeutic efficacy of mono- or combination therapies in MC38 tumors is largely resulted by adenosinergic antagonist-induced tumor death (Fig. 7j). The sparse presence of MC38-CAFs limits their direct interaction with TILs within the TME.

### The A_2B_ blockade-induced tumor regression is CAF-dependent

**Fig. 8:**
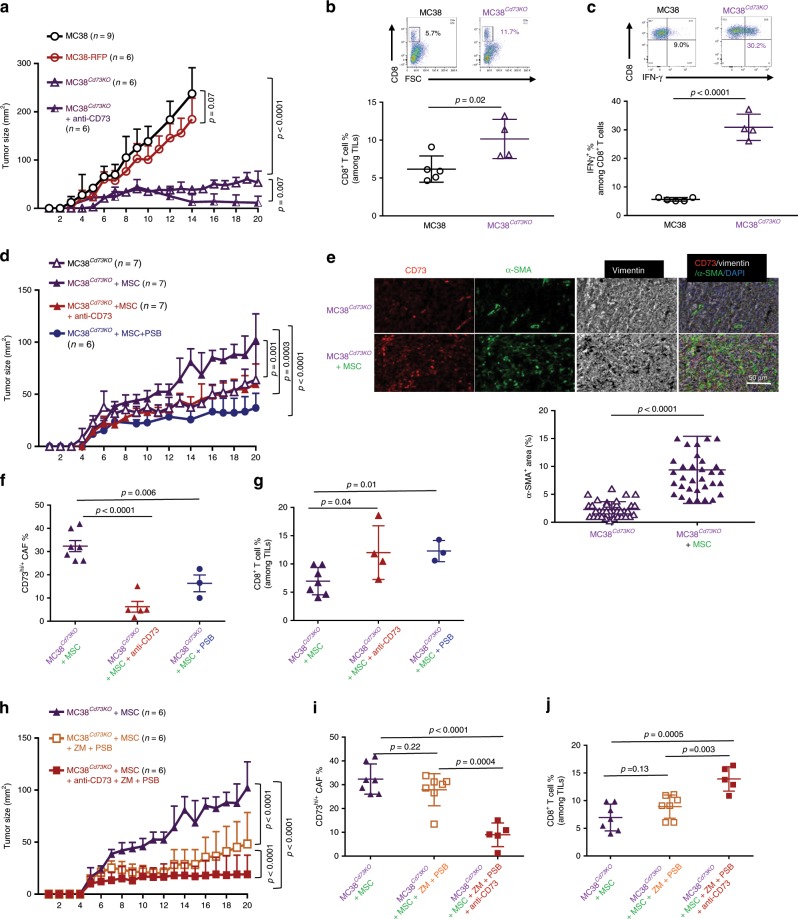
The synergistic effects of combined adenosinergic antagonism and CD73 blockade on tumor control is CAF-dependent. **a** The progression of MC38^*Cd73KO*^ and MC38-RFP in WT mice was compared the unmodified MC38 tumors. A group of MC38^*Cd73KO*^*-*bearing mice also received i.p. injection of anti-CD73 every other day. Tumor progression was examined every day. **b** The percentage of TIL-CD8 T cells and **c** IFN-γ-producing CTLs in the TME of MC38 and MC38^*Cd73KO*^ were determined via FACS and summarized. **d**–**g** The progression of MC38^*Cd73KO*^ tumors established in WT mice with MSCs co-inoculation with or without anti-CD73 or PSB1115 treatment was examined every day **d**. **e** CD73^+^ (red) and α-SMA^+^ (green) cells and their distribution within the TME of MC38^*Cd73KO*^ tumors without or with MSC co-inoculation were analyzed and quantified via IHC. **f**, **g** The percentage of CD73^hi/+^ CAFs (**f**) and TIL-CD8 T cells (**g**) in the TME MC38^*Cd73KO*^ tumors co-inoculated with MSCs following different treatments were determined via FACS and summarized. **h**–**j** The progression of MC38^*Cd73KO*^ tumors co-inoculated with MSC and treated with ZM243185 and PSB1115 combination in the absence or presence of anti-CD73 was examined every day **g**. **i**, **j** The percentage of CD73^hi/+^ CAFs **i** and TIL-CD8 T cells **j** in the TME MC38^*Cd73KO*^ tumors with MSC co-inoculation following different treatments were determined via FACS and summarized. **a**, **d**, **g** *p* values were determined via multiple *t*-tests where the tumor sizes at each time point were compared. **b**, **c**, **e**, **f**, **i**, **j** Error bars depict mean ± SEM. *p* values were determined via two-tailed unpaired Student’s *t*-test. All experiments were repeated at least two times. Source data are provided as a Source Data file.

To define whether the high CD73 expression in MC38 and/or their limited CAF distribution are the major barriers of effective combinatorial therapy, we generated MC38-*Cd73KO* single cell clones using the CRISPR/Cas9-directed gene knockout technology and FACSort based on the RFP-reporter expression. We used one clone, F2C11 (called MC38^*Cd73KO*^) for this study following the confirmation of *Cd73* inactivation/deletion in two alleles via surface staining, mRNA expression, and genomic DNA PCR flanking the target sequences at the exon-2 of *Cd73* gene (Supplementary Fig. 8a–c). Despite a comparable growth rate in culture between the MC38^*Cd73KO*^ and MC38 cells (Supplementary Fig. 8d), MC38^*Cd73KO*^ tumor progression in WT mice was significantly delayed compared to unmodified MC38 tumors and MC38 modified to express RFP (MC38-RFP) (Fig. 8a). The delayed MC38^*Cd73KO*^ progression was associated with a marked increase in tumor-infiltrating CD8 cells (Fig. 8b) and elevated IFN-γ production (Fig. 8c) without marked changes in either CAF abundancy or CD73^hi/+^ CAFs (Supplementary Fig. 8e). Importantly, CD73-neutralization further suppressed MC38^*Cd73KO*^ progression leading to their elimination in about 50% of the mice, suggesting the preservation of CD73-checkpoint during MC38^*Cd73KO*^ tumor progression (Fig. 8a).

Co-inoculation of MC38^*Cd73KO*^ with MSCs greatly accelerated tumor progression with a marked increase in CAF (α-SMA^+^) abundancy (Fig. 8d, e, Supplementary Fig. 8g, h), which marginally impacted TIL-CD8 cells (Fig. 8g, Supplementary Fig. 8g, h). CD73-neutralization or A_2B_ antagonist PSB1115 significantly suppressed MSC/CAF-assisted MC38^*Cd73KO*^ progression by inhibiting CAF-CD73 upregulation and enhancing TIL-CD8 cells (Fig. 8f, g) without marked alterations in CAF abundancy (Supplementary Fig. 8i). Combinatorial regimen of ZM241385 and PSB1115 markedly suppressed MSC/CAF-assisted MC38^*Cd73KO*^ progression, which was further enhanced by CD73-neutralization (Fig. 8h) with significant suppression of CAF-CD73^hi/+^ population and enhanced TIL-CD8 cells (Fig. 8i, j). Interestingly, ZM241385 and PSB1115 combination in the absence of anti-CD73 resulted in a marked reduction of CAFs, but not the frequency of CD73^hi/+^ CAFs (Fig. 8i, Supplementary Fig. 8j). Noticeably, neither combinatorial regimens impacted the frequency of IFN-γ^+^ CD8 T cells (Supplementary Fig. 8k). Together, in this modified MC38-CRC model, where tumor *Cd73* is inactivated with adequate presences of CAFs, the synergistic effects of adenosinergic antagonism and CD73-blockade was recapitulated as those seen in the EG7 tumor model. These results suggest that the abundancy and distribution of CAFs are essential for effective therapy of combined adenosinergic antagonism and CD73-neutralization.

## Discussion

**Fig. 9:**
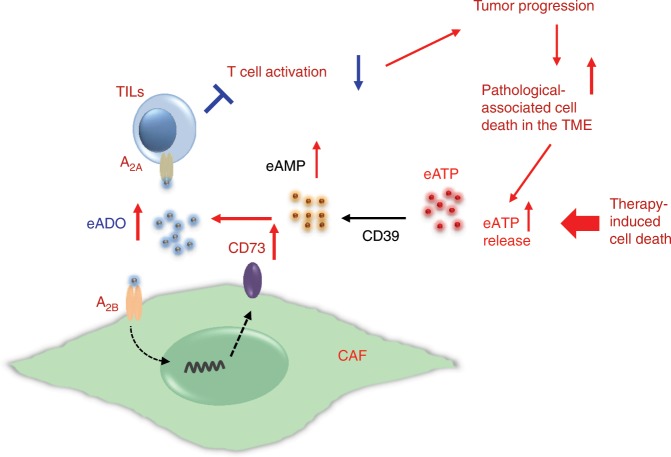
A schematic illustration of the proposed mechanism of pathological and therapy-induced CAF-CD73 upregulation enforcing the immune checkpoint in the TME.

Immune checkpoint inhibitors targeting the PD-1/PD-L1 or CTLA-4 pathway to unleash tumor-mediated immunosuppression remarkably improved clinical outcomes for some patients with various types of cancer^45,46^. Here, we elucidate that the highly elevated CD73 activity in CAFs by converting eATP to eADO and through the ADO-CD73 pathways in CAF-network represents another previously unidentified, non-redundant immune checkpoint. Our results substantiate the pivotal roles of CAFs in enforcing immunosuppression and promoting tumor progression, and shed light onto one of the mechanisms of action, which is by far not fully explicated^15,17,20,47,48,49–52^. Mechanistically, elevated ADO in the TME upregulates CD73 on CAFs via A_2B_-mediated pathway, thereby inciting the ADO-A_2B_-CD73 feedforward circuitry (Fig. 9), which further augments immunosuppression by activating the non-redundant ADO-A_2A_ pathway in immune cells to inhibit immune activation (Fig. 9). Importantly, our demonstrated A_2B_-mediated CD73 upregulation in CAFs and mesenchymal-like tumors provides not only a mechanistic explanation for previously reported therapy or stress-induced CD73 upregulation^10,26,42,44,53,54^, but also informative practical considerations for inhibiting immune checkpoint with minimal toxicity. Pathological or therapeutic-associated tissue damage/cell death may rapidly elevate eADO level up to 100 μM^29,55^, which strongly activates the A_2B_ pathway for CD73 upregulation regardless of A_2A_ blockade^10,42,44^. In this context, A_2B_ blockade with the CD73-neutralization to collaboratively alleviate the prevalent CD73-ADO checkpoint in CAF leads to improvement in clinical outcomes. Therapeutically, our results with two murine tumor models underscore the effectiveness of simultaneous inhibition of the non-redundant suppressive pathway of A_2A_ and A_2B_-dependent CD73-ADO immune checkpoint for improving antitumor immunity.

Clinical association between elevated CD73 expression and poor prognosis has been well documented in many tumor types^7,9,26–28,49,56^. The contribution of CD73 activity in Tregs and vasculature to this overall CD73-mediated immunosuppression has been well demonstrated^24,45–47^. Nevertheless, the contribution of CD73 activity in CAFs, which are prominent stroma population in the TME associated with poor survival for many tumor types^15–18,23,50^, remains poorly defined. Our comparative studies, using the murine CD73^-^EG7 tumor model, modified MC38^*dD73KO*^-CRC model, and correlative analyses of human CRC specimens and published datasets, clearly establish that the CD73^hi^-CAF identity represents a general attribute and a shared immunosuppressive mechanism among different tumor types and species. Our data thus support the previous work demonstrating that CD73 on CAFs promotes tumor immune escape in a mouse model of ovarian cancer^9^. Furthermore, our results demonstrate an association between elevated CD73 levels in the TME and CAF abundancy, poor clinical outcomes in CRC patients, and accelerated tumor progression in mice models.

Particularly, the synergistic effects of combinatorial adenosinergic antagonism and CD73-neutralization regimen largely depends on the abundant CAF distribution within the TME. Besides the sparse CAF distribution in the MC38 TME, the mesenchymal-like EMT characteristics of MC38, unlike the typical CD73^−^ CRC-adenocarcinomas, further complicated the therapeutic outcomes via: (1) a similar response to ADO-A_2B_-mediated CD73 upregulation to that observed in CAFs, which is potentially overwhelmingly active and counter-productive, especially at the sites of therapy-induced tumor death, contributing to the ineffective suppression of the overall CD73 levels/activity within the TME, and (2) an equally if not more detrimental attribute of the high susceptibility of MC38 to ZM243185-induced rapid and massive cell death, which triggers additional toxicity to the TILs and CAFs besides enforcing the ADO-A_2B_-CD73 circuitry (Fig. 7 and Supplementary Fig. 7). Therefore, for tumors that are highly sensitive to adenosinergic antagonism for apoptosis-induction and/or ADO-induced CD73 upregulation, A_2A_ and A_2B_ antagonists should be used with caution, whereas improving the dose and delivery route of anti-CD73 regimen will be safer. Undoubtedly, initial assessment of clinical patients/TME with specific considerations of the CAF abundancy, potential effects of ADO on tumor-CD73 levels, and their sensitivity to adenosinergic antagonist-induced cell death will further improve clinical outcomes and limiting toxicity for personalized immunotherapy and other therapeutic strategies to effectively inhibit CD73 checkpoint^57–59^.

In summary, this study clearly and robustly illustrates one of previously unidentified immunosuppressive functions of CAFs via high CD73 expression and bioactivity. This CAF-CD73^hi^ status is dynamically regulated and enforced during tumor progression and therapy via CAF/tissue-specific A_2B_ pathway. Therapeutically, simultaneous blockade of the non-redundant CAF-A_2B-_CD73 circuit and ADO-A_2A_-dependent inhibition of immune activation will be vital to improve the currently proposed/on-going tests of A_2A_ and/or CD73-neutralization clinical trials, as well as other activate therapies that induce massive cell death^10,26,42,60,61^, to alleviate the CAF-CD73-mediated immune checkpoint for ultimate and durable tumor control.

## Methods

### Mice, cell lines, and clinical specimens

*C57BL/6* mice were purchased from Charles River (Wilmington, MA). *CD73*^null^ (*B6.129S1-Nt5e*^*tm1Lft*^*/J*), GFP-Tg (*C57BL/6-Tg(UBC-GFP)30Scha/J*), and *A*_*2B*_^null^ (*B6.129P2-Adora2b*^*tm1Till*^*/J*) mice were obtained from the Jackson Laboratory (Bar Harbor, ME). All mice were maintained under specific pathogen-free conditions in the animal facility of Augusta University following protocols approved by the Augusta University Institutional Animal Care and Use Committee. Ova-expressing E.G7-OVA (EG7) cell line was purchased from ATCC (CRL-2113, Manassas, VA) and murine colorectal cancer cell line, MC-38, was obtained from Kerafast (ENH204-FP, Boston, MA). Early passage MSCs from *C57BL/6* background were purchased from Cyagen (MUBMX-01001, Santa Clara, CA). Mycoplasma contamination was periodically tested with the culture medium via the standard PCR-based assay. De-identified FEPE and frozen human colorectal cancer specimens were obtained from Georgia Cancer Center Biorepository following protocols approved by the Augusta University Institutional Review Board committee.

### Antibodies and reagents

Antibody staining for flow cytometry analysis of relevant markers was performed with 1 × 10^6^ cells in 100 μl staining buffer at the Ab concentration of 2 μg ml^−1^: anti-CD4 (RM4-5), anti-CD8α (53-6.7), anti-CD11b (M1/70), anti-CD25 (PC61), anti-CD31 (MEC13.3), anti-CD45.2 (104), anti-CD69 (H1.2F3), and anti-CD90.2 (53-2.1) were from BD Biosciences (San Jose, CA); anti-CD73 (TY/11.8), anti-IFN-γ (XMG1.2), and 7-AAD (7-Aminoactinomycin D) were from eBioscience (San Diego, CA); anti-mouse Gp38 (8.1.1) and Zombie Violet Fixable Viability Dye (#423113) were from Biolegend (San Diego, CA). Anti-HIF-1α (D2U3T) was from Cell signaling (Boston, MA); anti-ER-TR7 was from Abcam (Cambridge, MA); Alexa Fluor-conjugated goat secondary Abs were from Life Technologies (Carlsbad, CA). Functional-grade purified anti-CD73 (clone TY/23) and rat IgG2a isotype control (clone 2A3) for in vivo study were obtained from Bio X Cell (West Lebanon, NH). Mouse T-activator CD3/CD28 Dynabeads and CFSE [5(6)-carboxyfluorescein diacetate, succinimidyl ester] were from Life Technologies. Adenosine, AMP, and APCP were from Sigma (St. Louis, MO). Adnosinergic receptor antagonists, PSB1115 and ZM241385, were purchased from Tocris Bioscience (Bristol, UK). LSRII (BD Biosciences) was used for flow cytometry acquisition and data were analyzed using FlowJo (Tree Star Inc., Ashland, OR). The catalog number and concentration of each antibody used in this study is compiled in Supplementary Table 3.

### Tumor inoculation and treatment

EG7 and MC38 cells were maintained in DMEM (Invitrogen, Carlsbad, CA) with 10% FBS and 0.4 mg ml^−1^ G418. Tumor cells in exponential growth phase were collected and injected subcutaneously at 1 × 10^6^ mouse^−1^ in the right flank of *C57BL/6* WT or *CD73*^null^ mice. Tumors were measured every other day and calculated as: volume = (length × width). For BMT study, *C57BL/6* (or GFP-Tg) WT or *Cd73*^null^ mice were first irradiated with a JL Shepherd (San Fernando, CA) Mark 168 animal irradiator with a Co^60^ source for 950 cGy. Four hours following irradiation, each mouse received 5 × 10^6^ total bone marrow cells from either *C57BL/6* (or GFP-Tg) WT or *Cd73*^null^ mice via tail vein. Eight weeks following BMT, bone marrow chimerism of >95% donor origin circulating blood cells was confirmed via FACS. Therapeutic interventions, including anti-CD73 or adenosinergic antagonist treatment, were initiated as soon as s.c. inoculated EG7 tumors reached the size of ~20–25 mm^2^, usually around day 5 post-inoculation, by i.p. injection of 100 μg anti-CD73 or rat IgG2a isotype control every other day or daily i.p. injection of ZM241385 or PSB1115 at 1 mg kg^−1^ body weight.

### CAF purification and culture

EG7 tumor tissues pooled from multiple mice were dissected and minced into small pieces and digested with 0.8 mg ml^−1^ Dispase, 0.2 mg ml^−1^ Collagenase P, and 0.1 mg ml^−1^ DNase I (all from Roche, Basel, Switzerland) at 37 °C for 60 min with shaking. At the end of 60 min digestion, digested tissues were pipetted forcefully to disperse them into single cell suspension. These digested cells were FACSort purified using FACSAria with a 100 μm nozzle (BD Biosciences) as CD45^−^CD11b^−^CD31^−^GP38^+^ CAFs with a purity of >95%. These fresh CAFs were used for RNA harvesting and RNA-seq analysis. For in vitro CAF culture experiments, the above enzymatically digested cells were plated in 100-mm dishes in DMEM medium containing 10% FBS and non-adherent cells were removed 12 h later. The adherent cells, usually consisting of 30% CD11b^+^ cells and ~70% CD11b^−^CD45^−^ stromal cells, were subsequently purified by FACsorting as CD45^−^CD11b^−^CD31^−^GP38^+^ CAFs (>95% purity) or alternatively enriched via a magnetic bead-based negative depletion process using the EasySep mouse CD11b selection kit (Stemcell Technology, Vancouver, Canada) with a modification of adding additional anti-CD45.2 (clone 104) together with anti-CD11b from the EasySep kit to remove potential residual EG7 tumors and other immune cells. This usually resulted in a purity of > 90% CD45^−^CD11b^−^CD31^−^GP38^+^ CAFs. These purified CAFs were used immediately for in vitro experiments. Early passage (p6) MSCs from Cyagen were expanded for no more than two passages in DMEM medium with 10% FBS before being used for RNA-seq analysis or other experimental treatments as described.

### Tissue histology and IF staining

Human CRC Multiplex IHC staining was performed using sections of formalin-fixed paraffin embedded human CRC specimen and reagents in the Opal 7 solid tumor immunology kit (Perkin Elmer, USA) as per manufacturet's instructions. The antibodies used were listed as the following clones with dilutions listed in Supplementary Table 3: anti-α-SMA (1A4), anti-CD3 (SP7), anti-Vimentin (EPR3776), and anti-CD11b (EPR1344) from Abcam (Cambridge, MA), and anti-CD73 (D7F9A) from Cell Signaling (Danvers, MA). Images were acquired by using Vectra 3 quantitative pathology imaging system (Perkin Elmer). The image analysis and calculation of co-localization were performed using Inform software (Perkin Elmer) with 14–20 randomly selected and evenly distributed areas of ~5 × 10^5^ μm^2^ from each section. The relative percentage of CD73 distribution on CAFs, myeloid, and T cells was calculated against the total CD73^+^ region in each area, which was set as 100%. Tumor samples were snap frozen in liquid nitrogen. Cryosections were fixed in ice-cold acetone and pre-incubated in blocking solution (1% BSA and 5% goat serum in PBS) for 30 min at room temperature (RT). The primary antibody labeling was performed overnight at 4 °C, followed by a secondary antibody binding for 2 h at RT. Assessment of tumor hypoxia was performed using the Hypoxyprobe^TM^ kit (Hypoxyprobe Inc., MA). Specifically, tumor-bearing mice received i.p. injection of pimonidazole HCl in PBS at 60 mg kg^−1^ 90 min before euthanasia. Tumors were immediately excised and snap frozen in liquid nitrogen. Cryosections were fixed in ice-cold acetone and pre-incubated in blocking solution for 30 min at room temperature (RT) and a subsequent incubation with anti-pimonidazole antibody (MAb1, clone 4.3.11.3) at 1.2 μg ml^−1^ overnight at 4 °C, followed by a secondary antibody binding for 2 h at RT. Images were acquired using Evos (Life Technologies) fluorescent microscope and subsequently resized using Adobe Photoshop CS.

### Principle component analysis (PCA) of gene expression

We first defined FB-specific or immune response-associated genes. The 18 FB-specific genes were selected based on highly elevated genes in CRC-CAFs reported by Calon et al. (*Nat. Genet.* 47, 320–329 (2015)). Most of the 18 selected genes are those commonly used to define CAFs or FBs. The 15 immune response-associated genes were generated from the commonly used immune signaling and function-related genes. The specificity of these two sets of pre-defined genes were further validated by at least two-fold higher expression in purified CRC-CAFs or CRC-TILs, respectively, compared with purified counterparts in the same TME from two published datasets (GSE39396 and GSE39395). The gene expression dataset (GSE39582) of a cohort of 585 human CRC specimens was used for PCA to first extract the *NT5e*^high^ and *NT5e*^low^ groups as specimens in the top 20% and bottom 20% of *NT5e* expression levels, respectively. They were subsequently clustered with *K*-means (*K* = 4) according to the category of *NT5e*^high^ and *NT5e*^low^ expression against their average extents of the overall normalized expression of the pre-defined FB-specific genes, which revealed a positive correlation. Likewise, the *K*-mean clustering was applied to the category of *NT5e*^high^ and *NT5e*^low^ versus the 15 pre-defined immune response-associated genes established a negative correlation. Finally, the overlapping 106 CRC specimens were generated for plotting the centroid PCA analyses on *NT5e*^high^ and *NT5e*^low^ against either the expressions of FB-specific genes or that of immune response genes with the successfully separated clusters corresponding to the levels of *NT5E* expression as shown in Fig. 1.

### High performance liquid chromatography (HPLC) assay for adenosine

HPLC was performed using the Prominence UFLC liquid chromatography system (Shimadzu, Toyko, Japan) with a 2.1 × 100 mm Ascentis Express OH5 column (Sigma-Aldrich). Mobile phase A consisted of 10% 10 mM ammonium acetate buffer adjusted to pH 5.5 in 90% acetonitrile. Mobile phase B consisted of 50% acetonitrile. A linear gradient profile from 15% B (5 min) to 100% B (1 min) followed by a 5 min period of 15% B. The column temperature was controlled at 33 °C and UV260 nm was used for adenosine detection. The LC system was controlled and the output signal was monitored by using Shimadzu LC solution software.

### CD8^+^ T cell activation in CAF condition medium

Purified CAFs were seeded in a six-well plate at 1 × 10^5^ well^−1^. After the overnight culture for CAF attachment, 200 μM AMP was added for 4 h and supernatant harvested as CAF condition medium (CAF-CM). Naïve CD8 T cells were enriched using EasySep^TM^ negative selection kit (Stem Cell Technology). 5 × 10^5^-purified T cells, either CFSE-labeled or unlabeled, in fresh RPMI-1640 medium (10% FBS) were mixed with CAF-CM at 1:1 and activated by α-CD3/CD28 activation beads (Life Technologies). Early T cell activation was analyzed for 6–15 h post-stimulation and T cell proliferation as CFSE dilution was examined 2–3 days post-stimulation.

### Analyses of gene expression via RNA-seq and RT-PCR

CAFs were purified from EG7 tumor-bearing mice as described above. Early passage (p6) murine MSCs purchased from Cyagen were cultured in DMEM supplemented with 2 mM of l-glutamine, 3.7 g/l NaHCO_3_, and 10% FBS for no more than two passages. Total RNA was harvested from purified CAFs and MSCs using Trizol reagent (Thermo Fisher Scientific) followed with Purelink RNA mini kit (Thermo Fisher Scientific). RT was performed with 1 μg of total RNA using random hexamers as primers and Superscript II reverse transcriptase (Life Technologies). Real-time PCR was carried out following standard protocols with primers purchased from Integrated DNA Technologies (Coralville, IA) using a BioRad CFX384 (BioRad Life Science Research, Hercules, CA). Primers used for the amplification of murine *Nt5e, Hif1a, vegfa, Adora2a, Adora2b*, and *β-actin* are listed in Supplementary Table 2.

For RNA-seq library preparation, ~200–300 ng Total RNA was used following the Illumina TruSeq stranded mRNA sample preparation guide. After RNA-seq libraries were subjected to quantification process, pooled for cBot amplification and subsequent 50 bp single read sequencing run with Illumina HiSeq 3000 platform. After the sequencing run, demultiplexing with Bcl2fastq2 was employed to generate the fastq file for each sample.

RNA quality assessment, libraries preparation, and RNA sequencing were all performed in the Genome Core of Greehey Children’s Cancer Research Institute, University of Texas Health Science Center at San Antonio. High throughput RNA sequencing reads were mapped to the reference genome (NCBI, UCSC/mm10) using Tophat 2.0.11^62^ and the relative gene expression was estimated using cufflinks 2.0.2^63^. All RNA-seq data are available at Genes Expression Omnibus (GEO) under the accession number GSE141199. Gene expression differential testing was performed by using cuffdiff 2.0.2^63^. Genes with adjusted *p* < 0.01 were selected as significantly differentially expressed. GSEA4.0.0 software (Broad Institute) was used for GSEA and KEGG pathway analysis was performed using WEB-based GEne SeT AnaLysis Toolkit (http://www.webgestalt.org/option.php). Heat maps of DEGs were created using MORPHEUS online software.

### Human patient sample analysis

Transcriptome datasets of human CRC and breast cancer patient specimens were downloaded from NCBI GEP or the cBio Cancer Genomics Portal website. The transcriptomic profile, expression of *NT5E*, *HIF1A*, and *EPAS1*, gene coexpression network analysis, and Kaplan–Meier curve of patient survival probability were carried out following standard procedures, including the R2: Genomics Analysis and Visualization Platform (http://r2.amc.nl).

### CRISPR/Cas9-based *Nt5e* gene knockout in MC38 tumors

*Cd73* CRISPR/Cas9 KO plasmids (Santa Cruz, sc-423919) and *Cd73* HDR plasmids (Santa Cruz, sc-423919-HDR) were co-transfected into MC38 tumors using Invitrogen Lipofectamine LTX transfection reagent. Forty-eight hours post-transfection, transfected MC38 cells were first subjected to a 5-day puromycin selection followed by single cell cloning via FACSorting of RFP^high^ cells. Following expansion of various MC38^*Cd73KO*^ clones, their expression of reporter RFP associated with the lost CD73 expression in culture and in vivo were confirmed via FACS analysis. Subsequently, total RNAs and genomic DNA were harvested from these established MC38^*Cd73KO*^ clones for further confirmation as the molecular levels for their lack of *Cd73* mRNA expression and deletions via PCR-based amplification with primers annealing exon 2 region of the CD73 gene, which was suggested by the Santa Cruz Technology technical support personnel as one of the regions flanked by targeted constructs for *Cd73* deletion. For in vivo studies, 5 × 10^5^ MC38^*Cd73KO*^ cells without or with 2 × 10^5^ MSCs were suspended in 0.2 ml of PBS and inoculated s.c. into the flank of WT mice. Tumors were measured every other day. Five days post-tumor inoculation when tumors were palpable, different groups of mice received various treatments of either monotherapy of individual adenosinergic antagonist, anti-CD73, or combinatorial therapy of both adenosinergic antagonism and CD73 neutralization as described above.

### Statistical analysis

Statistical significance was assessed by two-tailed unpaired nonparametric or Student’s *t*-test using Prism 7 software (GraphPad, CA). A *p* value of <0.05 was considered significant and was indicated in each figure. Statistical differences in tumor progression between two groups of experimental mice was analyzed via multiple *t*-test at each tumor measurement time point using Prism 7. The specific *p* value with relevant statistical analysis used for each study is presented in the figure panel.

### Reporting summary

Further information on research design is available in the Nature Research Reporting Summary linked to this article.

## Supplementary information

Supplemental Information

Reporting Summary

## Data Availability

The RNA-seq data that support Fig. 3 of this study are available at Genes Expression Omnibus (GEO) under the accession number GSE141199 (https://www.ncbi.nlm.nih.gov/geo/query/acc.cgi?acc=GSE141199). All data, including those for supplementary figures, are provided in the compiled Source Data files.
