# Supplementary material for: CD73 on cancer-associated fibroblasts enhanced by the A2B-mediated feedforward circuit enforces an immune checkpoint
Source: Nat Commun. 2020 Jan 24;11:515. doi: 10.1038/s41467-019-14060-x (PMC6981126; doi:10.1038/s41467-019-14060-x)
Supplement: Supplementary file 1 — Supplemental Information [file 41467_2019_14060_MOESM1_ESM.pdf]

SUPPLEMENTARY INFORMATION

CD73 on cancer-associated fibroblasts enhanced by the A_{2B}-mediated feedforward circuit enforces an immune checkpoint

Yu et al.

List of supplementary materials

Supplementary table 1: Real-time RT-PCR primers
Supplementary table 2: PCR primers for genomic DNA amplification and confirmation of CD73 knockout
Supplementary table 3: List of antibody source and specific information used for flow cytometry and multiplex IHC staining

Supplementary Figure 1. High CD73 levels in the human CRC TME correlate with increased CAFs and poor immune response profile.
Supplementary Figure 2. CAFs in the murine TME are $CD73^{hi}$ representing the major source of eADO generation.
Supplementary Figure 3. CAFs are transcriptionally modulated in a similar pattern in the murine and human TME.
Supplementary Figure 4. *Cd73* inactivation in the TME of $Cd73^{null}$ mice enhances effector T cell activation.
Supplementary Figure 5. CAF-CD73 expression in the TME is progressively enhanced via A_{2B} activation by hypoxia or therapy-induced eADO.
Supplementary Figure 6. Differential effects of CD73 neutralization and A_{2B} or A_{2B} antagonist on CD73 expression and CAF abundancy in the EG7 TME.
Supplementary Figure 7. CD73 neutralization in combination with A_{2B} and A_{2B} antagonists in the MC38 TME modestly suppressed tumor progression through antagonism-induced tumor apoptosis.
Supplementary Figure 8. $MC38^{Cd73KO}$ tumors engineered via CRISPR/Cas9-based knockout system in the presence of adequate fibroblastic stroma recapitulate the collaborative therapeutic effects of adenosinergic antagonism and CD73-neutralization.

Supplementary Table 1: Real-time RT-PCR primers

Gene symbol	Forward primer	Reverse primer
β-actin	GAC TCA TCG TAC TCC TGC TTG	GAT TAC TGC TCT GGC TCC TAG
Nt5e	TGC CAC CTC CGT TTA CAA TG	GAA ACC TGA TCT GTG ATG CCA
Hif1a	CCG TCA TCT GTT AGC ACC AT	GCT CAC CAT CAG TTA TTT ACG TG
Vegfa	ATC ACT TCA TGG GAC TTC TGC	GAA CTT TCT GCT CTC TTG GGT
Adora2a	CTC ATA CCC GTC ACC AAG C	GAG TTC CAT CTT CAG CCT CTT
Adora2b	GTC CCA GTG ACC AAA CCT TT	ACA CAG AGC TCC ATC TTT AGC

Supplementary Table 2: PCR primers for genomic DNA amplification and confirmation of *Cd73* knockout

Gene symbol	Forward primer	Reverse primer
N/A- input control	CTC TGC TGC CTC CTG GCT TCT	CGA GGC GGA TCA CAA GCA ATA
Nt5e-exon 2	ATG TGA ATA AGA TCA TCG CC	CAT CAT ATA CTG CAC ACT GA

Supplementary Table 3: List of antibody source and specific information used for flow cytometry and multiplex IHC staining

Antibody	Assay	Source	Catalogue #	Dilution
CD4-PerCp/Cy5.5	FACS	BD Biosciences	550954	2 μg ml^{-1}
CD8α-APC	FACS	BD Biosciences	553035	2 μg ml^{-1}
CD11b-FITC	FACS	BD Biosciences	553310	2 μg ml^{-1}
CD25-PE	FACS	BD Biosciences	553866	2 μg ml^{-1}
CD31-APC	FACS	BD Biosciences	551262	2 μg ml^{-1}
CD45.2-PE	FACS	BD Biosciences	560659	2 μg ml^{-1}
CD69-FITC	FACS	BD Biosciences	553236	2 μg ml^{-1}
CD90.2-APC	FACS	BD Biosciences	553007	2 μg ml^{-1}
IFN-γ-PE/Cy7	FACS	eBioscience	25731182	2 μg ml^{-1}
7-AAD	FACS	Biolegend	420404	5 μl $sample^{-1}$
CD73-Alexa647	FACS	Biolegend	127208	2 μg ml^{-1}
Gp38-PE/cy7	FACS	Biolegend	127412	2 μg ml^{-1}
Zombie Violet Fixable Viability Dye		Biolegend	423113	1 μl $sample^{-1}$
ER-TR7	IF and IHC	Abcam	Ab51824	1: 100
α-SMA (1A4)	IF and IHC	Abcam	Ab7817	1: 300
CD3 (SP7)	IF and IHC	Abcam	Ab16669	1: 200
Vimentin (EPR3776)	IF and IHC	Abcam	Ab92547	1: 2000
CD11b (EPR1344)	IF and IHC	Abcam	Ab133357	1: 4000
CD73 (D7F9A)	IF and IHC	Cell signaling	13160	1: 200
Mouse CD8	IF and IHC	Abcam	Ab209775	1: 4000
EpCam	IF and IHC	Cell signaling	93790	1: 100
CD73 (TY/23, functional grade)	Neutralization	Bio X cell	BE0209	100 μg $injection^{-1}$
Rat IgG2a isotype control		Bio X cell	BE0089	100 μg $injection^{-1}$

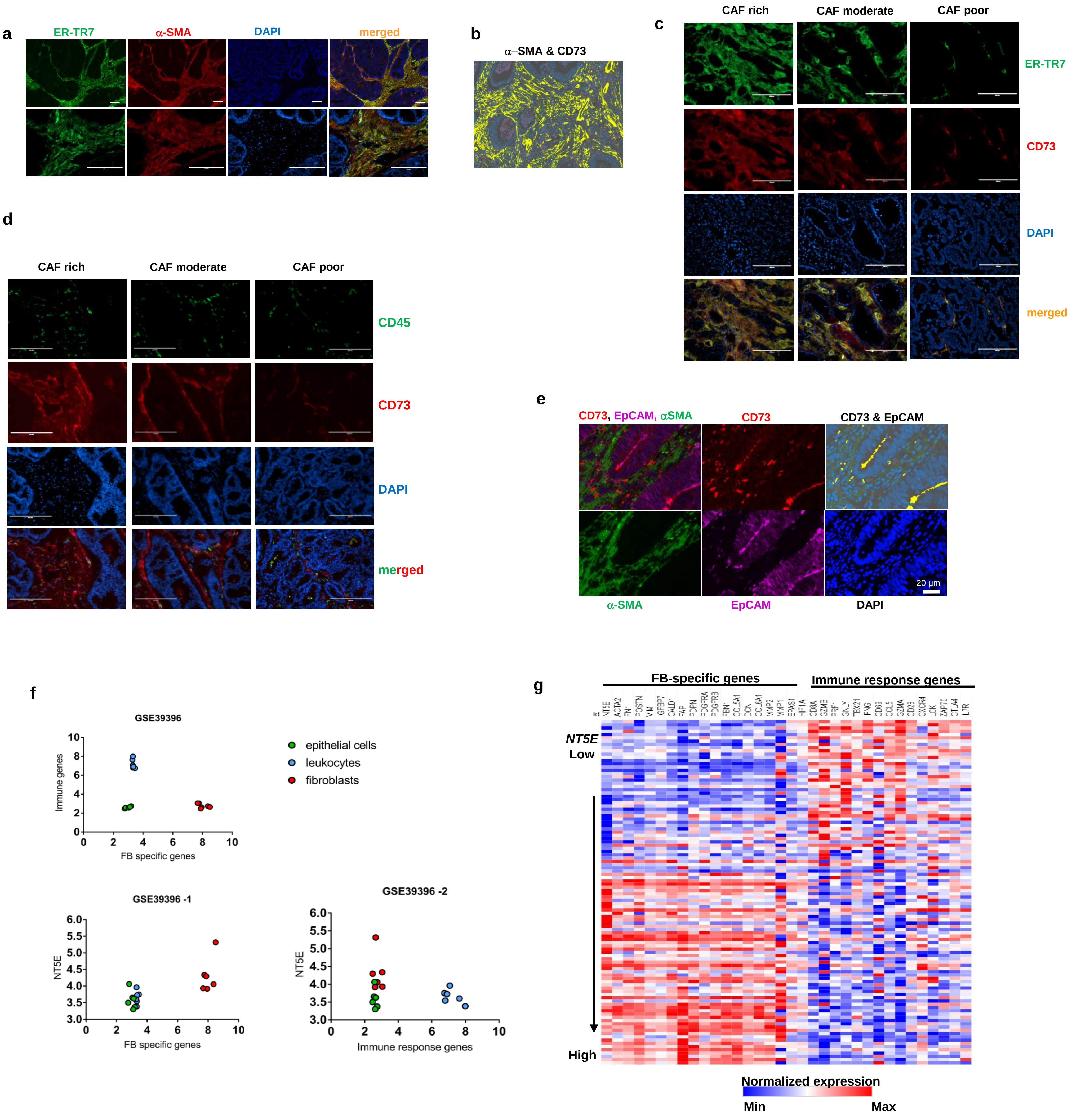

Supplementary Figure 1. High CD73 levels in the human CRC TME correlate with increased CAFs and poor immune response profile. (a) IF staining of ER-TR7 (green) and α-SMA (red) signals in human CRC specimens. Nuclei were counterstained with DAPI (blue). (b) A representative CRC image of multiplex IHC stained for CD73, α-SMA, CD11b, and CD3 and processed with PerkinElmer Inform software to identify the area of co-localization between CD73 and α-SMA (highlighted as yellow). (c, d) IF-staining to examine the bio-distribution of CD73^{+} (red) signal with ER-TR7^{+} (green) stromal cells (c) and CD45^{+} (green) immune cells (d) in stroma-rich, moderate, and poor CRC specimens. Scale bars, 200 μm. (e) Representative multiplex-IHC staining images showing the distribution of EpCAM (magenta), CD73^{+} (red), and α-SMA^{+} (green) signals with limited CD73 and EpCAM co-localization, which is mostly limited to the lumina. (f) Validation of the specificity of our defined 18 fibroblast-specific genes and 15 Immune response-specific gene expression pattern of the published CRC dataset (GSE39395), which shows distinct separation of CAFs from tumors and TILs based on these two gene-lists, as well as the relative relationship with CD73 expression in each cellular subset. (g) Expression profile of the 106 CRC specimens identified as NT5E^{high} and NT5E^{low} is presented for their differential expression pattern of the18-FB specific and 15-Immune response genes. Source data are provided in the Source Data file.

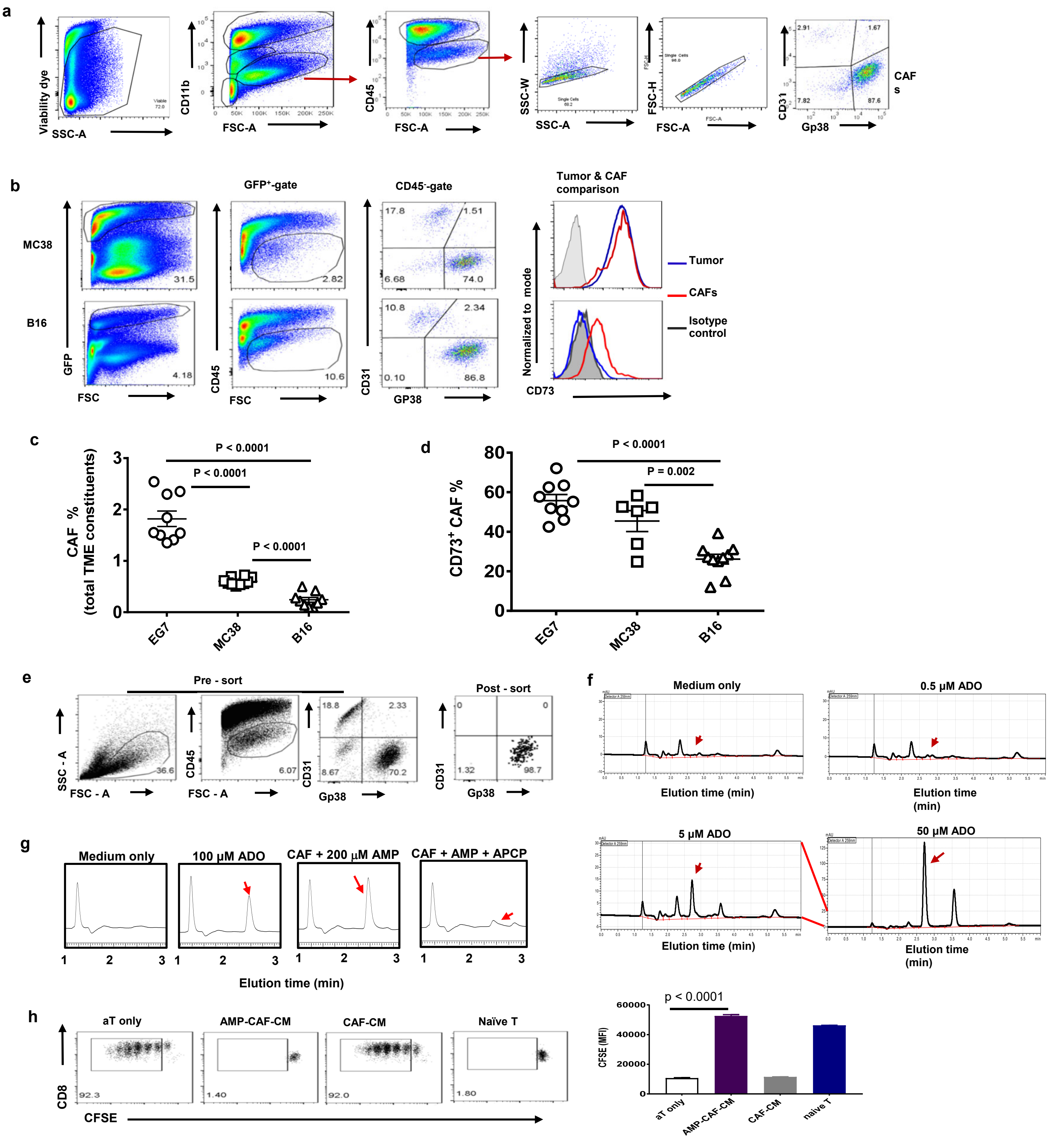

Supplementary Figure 2. CAFs in the murine TME are CD73^{hi} representing the major source of eADO generation. (a) Gating strategy for CAF assessment, defined as CD45$^-$GP38$^+$CD31$^-$ population, in the EG7 TME and for FACSort purification for results presented in Figs 2 - 6. (b) GFP-transgenic mice (*GFP-Tg*) were used as hosts to establish MC38 and B16 tumors s.c.. The frequency and phenotype of their CAFs were determined via FACS as GFP$^+$CD45$^-$GP38$^+$CD31$^-$ cells. Their levels of CD73 expression were compared with GFP$^-$ tumors. The percentage of CAFs (c) and *CD73*$^+$ CAFs (d) in the TME of MC38 and B16 were evaluated by FACS and compared with those in the EG7 TME. (e) EG7-CAFs were purified via FACSort. (f) Representative HPLC chromatograms for detecting various concentrations of ADO. (g) Quantification of the capacity of EG7-CAFs in ADO generation from 200 μM AMP in the absence or presence of CD73 inhibitor APCP. (h) CFSE labelled CD8 T cells were activated by α-CD3/α-CD28 beads in CAF-conditioned medium, pre-incubated with 200 μM AMP (AMP-CAF-CM) or without AMP (CAF-CM), for 3 days. T cell proliferation as CFSE dilution was examined via FACS. Statistical analysis and p value calculation were performed with Graphpad Prism 7 using two-tail, unpaired Student's *t*-test. All experiments were performed at least two times. Source data are provided in the Source Data file.

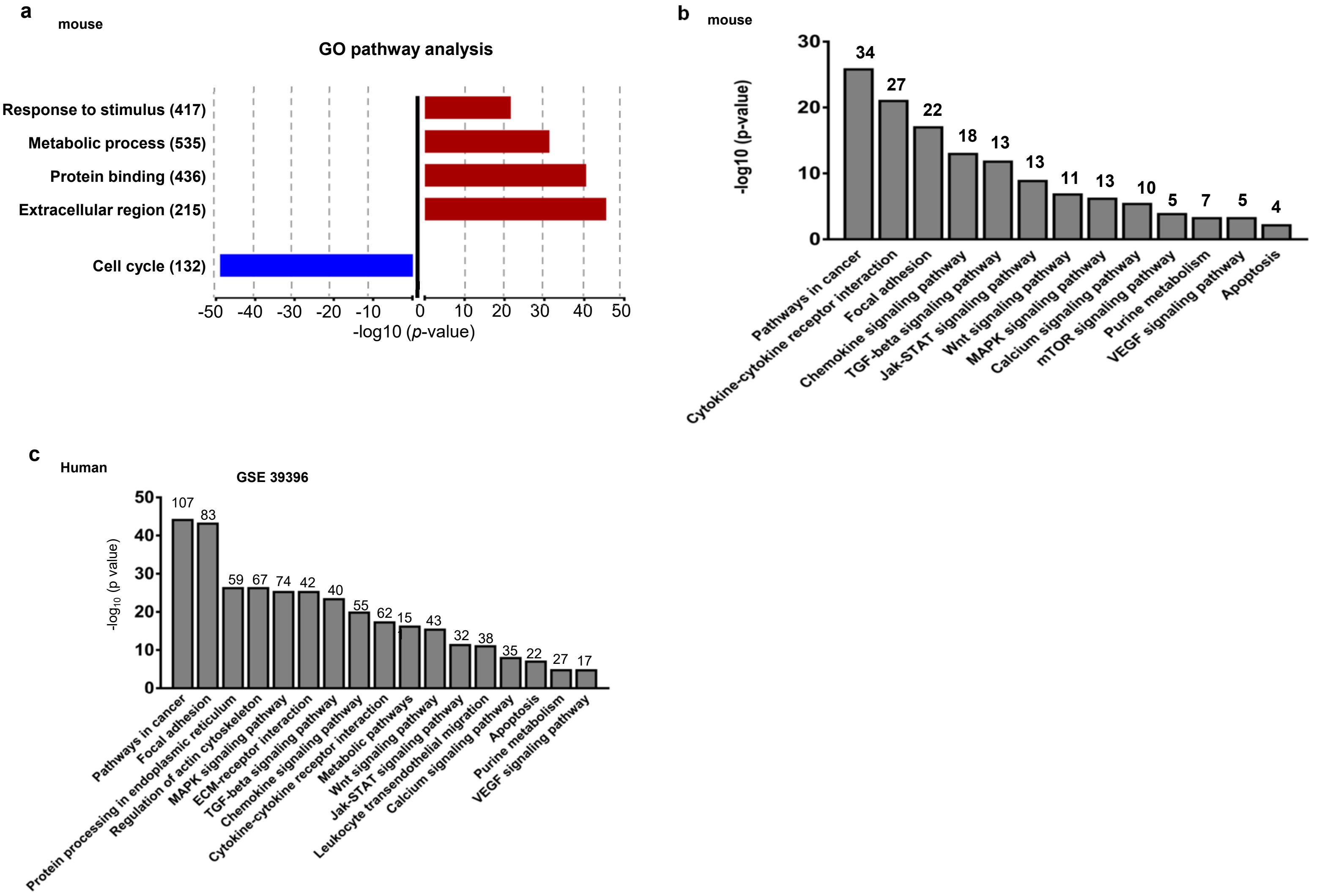

Supplementary Figure 3. CAFs are transcriptionally modulated in a similar pattern in the murine and human TME. (a) RNA-seq comparative analysis of EG7-CAFs and murine MSCs were performed. Gene Ontology (GO) pathway analysis was employed to identify the significantly up- and down-regulated genes for key biological pathways with highly enriched differentially expressed genes with the adjusted -Log$_{10}$ *p*-value, which indicates the significant of enrichments. (b) The 417 genes categorized in “response to stimulus” were further analyzed for enrichment in KEGG pathways. The number of genes significantly up-regulated in CAFs according to specific pathways were shown on top of each bar with corresponding level of significance. (c) KEGG pathway analysis of purified human CRC-CAFs and CRC-tumors from dataset GSE 39396 identified 2927 significantly up-regulate DEGs in CAFs. The number of genes associated with specific pathways were shown on top of each bar with corresponding level of significance as -Log$_{10}$ *p*-value. Source data are provided in the Source Data file.

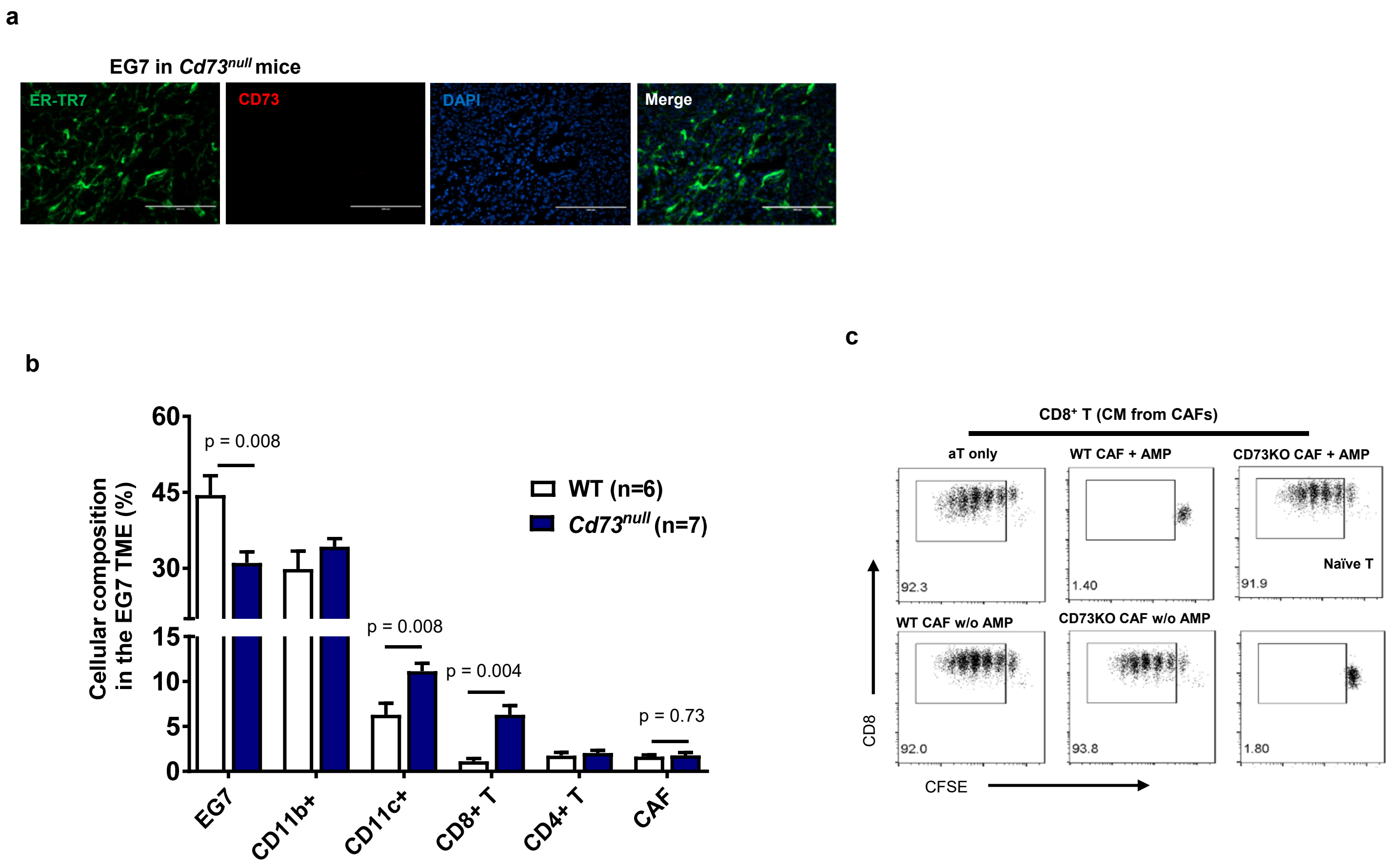

Supplementary Figure 4. *Cd73* inactivation in the TME of *Cd73*^{null} mice enhances effector T cell activation. (a) IHC staining of CD73 (red) expression in correlation to ER-TR7^{+} (green) CAFs in the EG7 tumor established in *Cd73*^{null} mice 9 days post-tumor inoculation. (b) Comparative analysis of cellular composition within the EG7 TME of WT and *Cd73*^{null} mice was summarized. (c) The effects of WT and *Cd73*^{null} CAF-conditioned medium on proliferation of CFSE labelled CD8 T cells following α-CD3/α-CD28 bead-induced activation was examined via FACS. Some of the CAFs were pre-incubated with 200 µM AMP (AMP-CAF-CM) or without AMP (CAF-CM). Statistical analysis and p value calculation were performed with Graphpad Prism 7 using two-tail, unpaired Student's *t*-test. Error bars depict mean ± SEM. All experiments were performed at least two times. Source data are provided in the Source Data file.

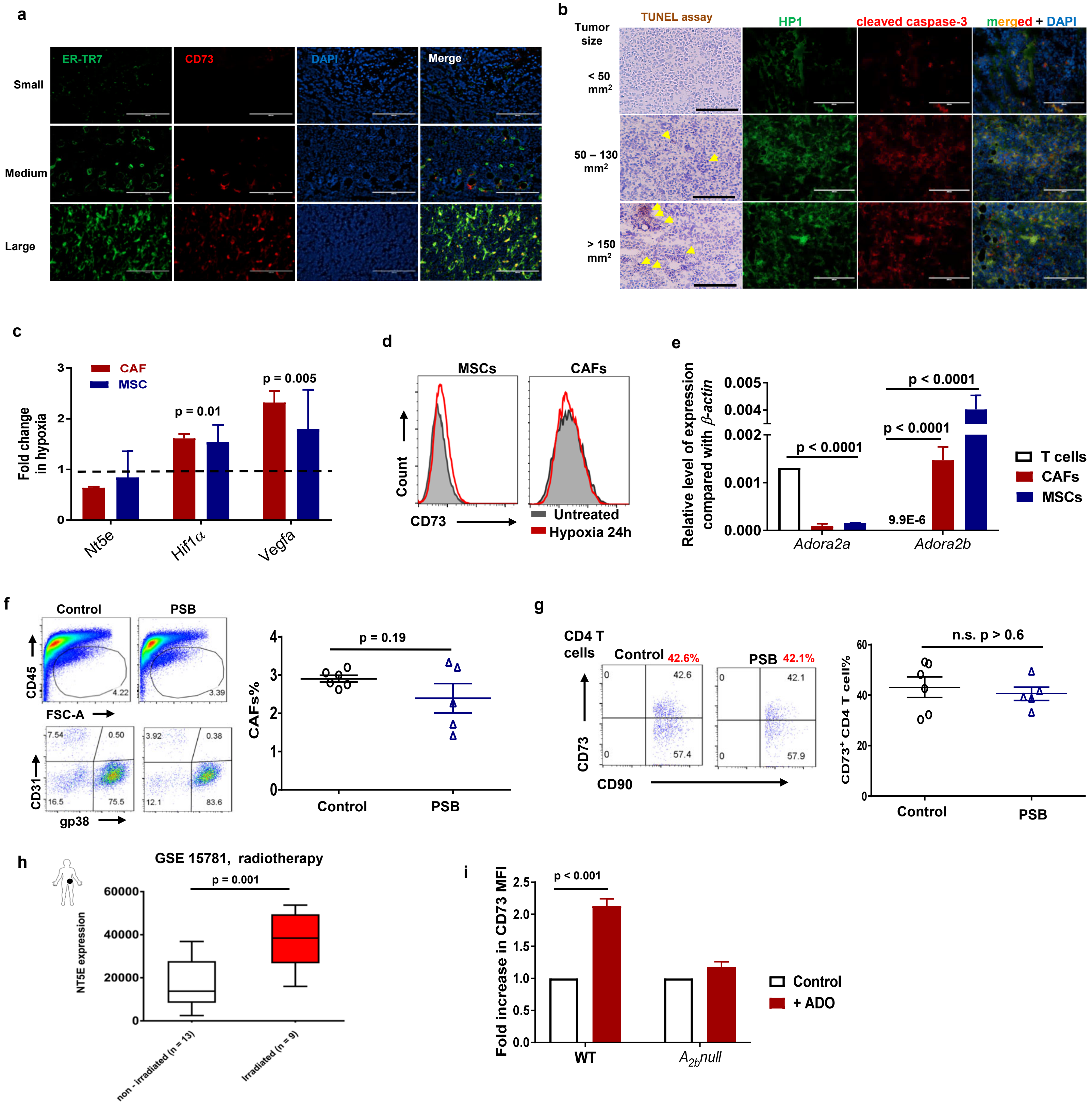

Supplementary Figure 5. CAF-CD73 expression in the TME is progressively enhanced via A_{2B} activation by hypoxia or therapy-induced eADO. (a) IF-staining examination of CD73 expression (red) on EG7-CAFs (ER-TR7⁺, green) in tumors of various sizes was shown as representative photograms. (b) Tumor necrosis, hypoxia, and apoptosis during EG7 progression in tumor of various sizes were assessed via IHC and IF staining using specific antibodies against DNA breaks (brown), the Hypoxyprobe^{TM}-1 (HP1) (green), and cleaved caspase-3⁺ cells (red), respectively. Scale bars, 200 μm. (c) EG7-CAFs and MSCs were cultured in a hypoxia chamber containing 1% O_2 for 6 hours. The expression of CD73 (*Nt5e*) and other hypoxia related genes was examined via quantitative real-time RT-PCR and compared with untreated CAF or MSC control, respectively, which was set as 1.0. (d) CAFs and MSCs were cultured in hypoxia chamber for 24 hours and their surface CD73 expression were examined via FACS. (e) Comparative analysis of A_{2a} (*Adora2a*) and A_{2b} (*Adora2b*) expression in purified naïve T cells, CAFs, and MSCs was performed via real-time RT-PCR. (f-g) EG7-CAF abundancy (f) and CD73 expression on CD4 T cells (g) in tumor bearing WT mice treated with daily i.p. injection of PSB1115 were examined via FACS. (h) *NT5E* (*CD73*) expression of a published dataset (GSE 15781) of CRC specimens obtained from patients treated without or with radiotherapy was analyzed. (i). EG7-CAFs from WT and A_{2b}^{null} mice were purified and cultured in the absence or presence of 100 μM ADO for 24 H. CD73 expression levels were examined by FACS and normalized against each untreated control, which was set at 1. Unpaired Student's t-test, (n = 3 - 4). Source data are provided in the Source Data file.

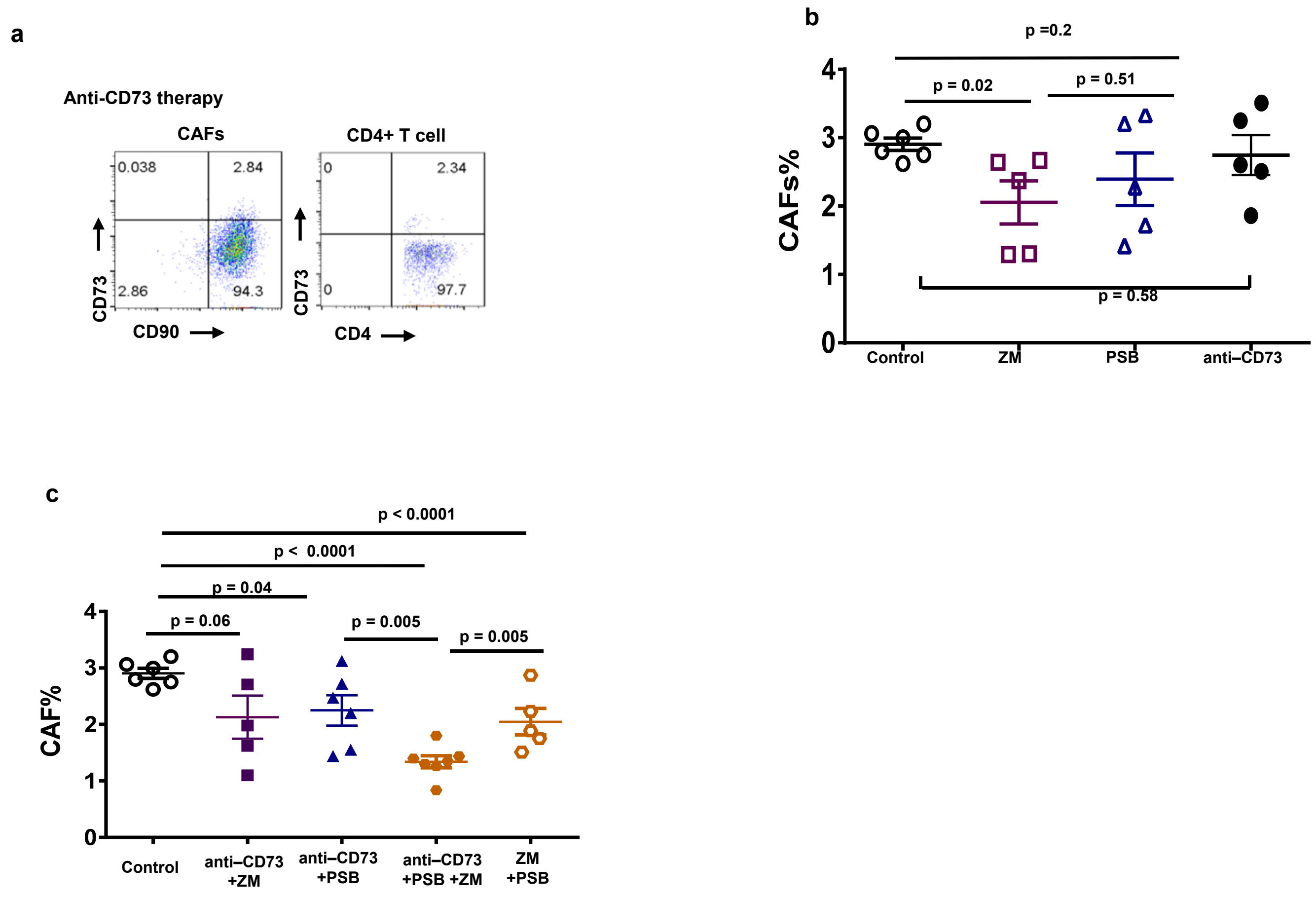

Supplementary Figure 6. Differential effects of CD73 neutralization and A_{2B} or A_{2B} antagonist on CD73 expression and CAF abundancy in the EG7 TME. (a) CD73 levels on CAFs and CD4⁺ T cells within the EG7 TME of mouse received anti-CD73 treatment was examined via FACS. Representative FACS plots of CD73 levels are shown. (b) The percentage of CAFs in the EG7 TME of mice treated with either ZM241385, PSB1115, or anti-CD73 was examined via FACS. (c) The percentage of CAFs in the EG7 TME of mice treated with combination therapy of ZM243185, PSB1115, or ZM243185 + PSB1115 with or with without anti-CD73 was analyzed via FACS and summarized. Source data are provided in a Source Data file.

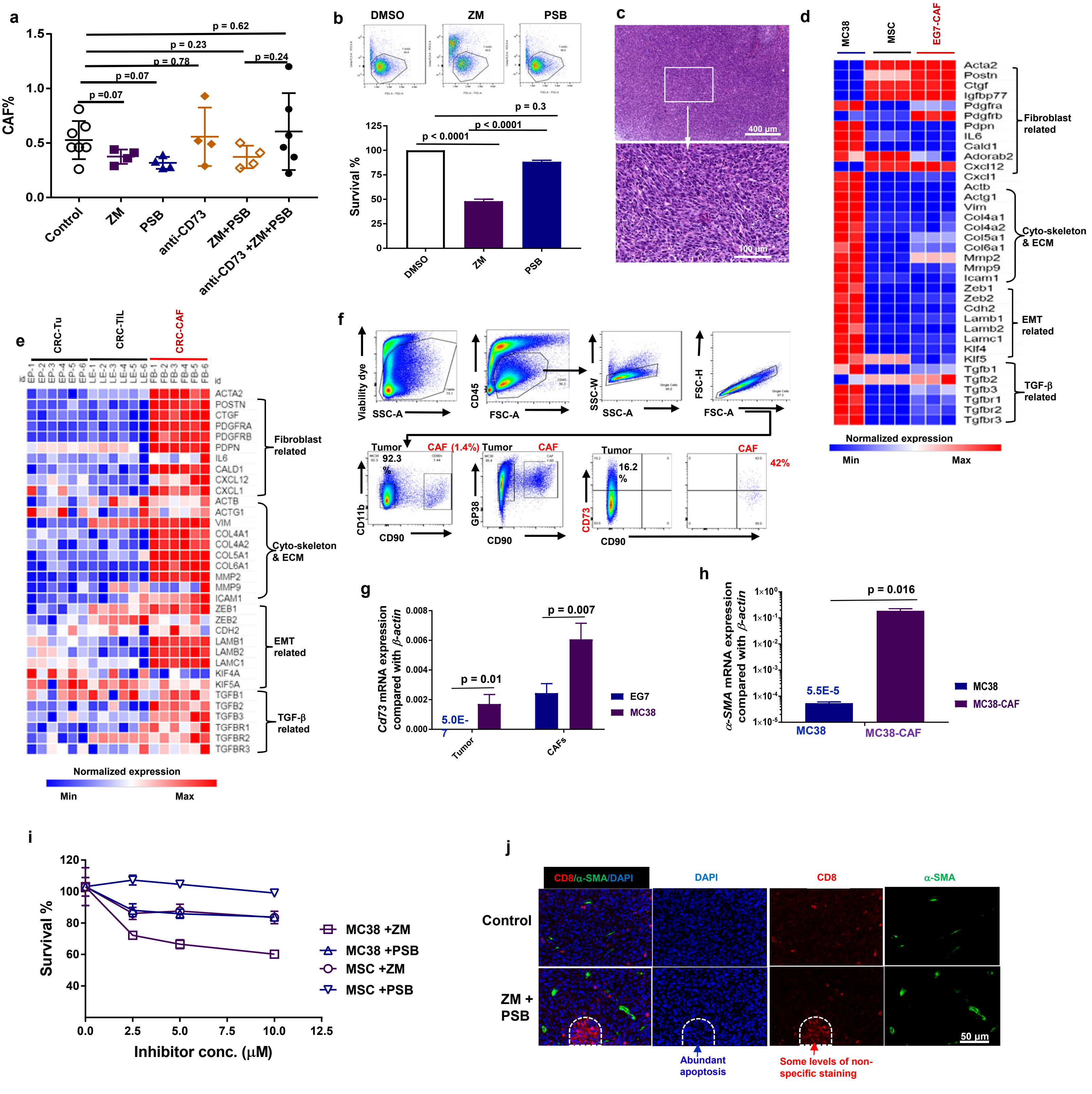

Supplementary Figure 7. CD73 neutralization in combination with A_{2B} and A_{2B} antagonists in the MC38 TME modestly suppressed tumor progression through antagonism-induced tumor apoptosis. (a) The percentage of CAFs in the MC38 bearing mice treated with ZM243185, PSB1115, anti-CD73 or combination therapy was analyzed via FACS. (b) MC38 tumors were treated with either 10 μM ZM241385 or PSB115 in culture for 48 H. The surviving cells were evaluated by FACS and cell counts. (c) Representative H&E images of established MC38 tumor for histological examination. (d) MC38 transcriptome profile (GSE112252) was compared with those of MSCs and EG7-CAFs with specific emphasis on gene expression pattern related to EMT. (e) Comparative transcriptome profile analysis of paired purified CR-tumors, CFAs, and TILs of GSE39396 dataset. (f) Gating strategy of FACSort purification of MC38-CAFs for subsequent comparative studies for results presented in Figures 7-8 and supplementary Figures 7 - 8. (g,h) Comparative real-time RT-PCR analysis of MC38-CAF and tumor gene expression with those of EG7 tumors. (i) Comparative analysis of the survival population of MC38 tumor and MSC following 48 H culture in the presence of various concentrations of ZM241385 or PSB115 using the ATPLite assay kit. (j) Representative IHC images showing CD8 T cell and CAF distribution in MC38 tumors treated without or with 10 μM ZM241385 + PSB115. Scale bars: 50 μm. Source data are provided in the Source Data file.

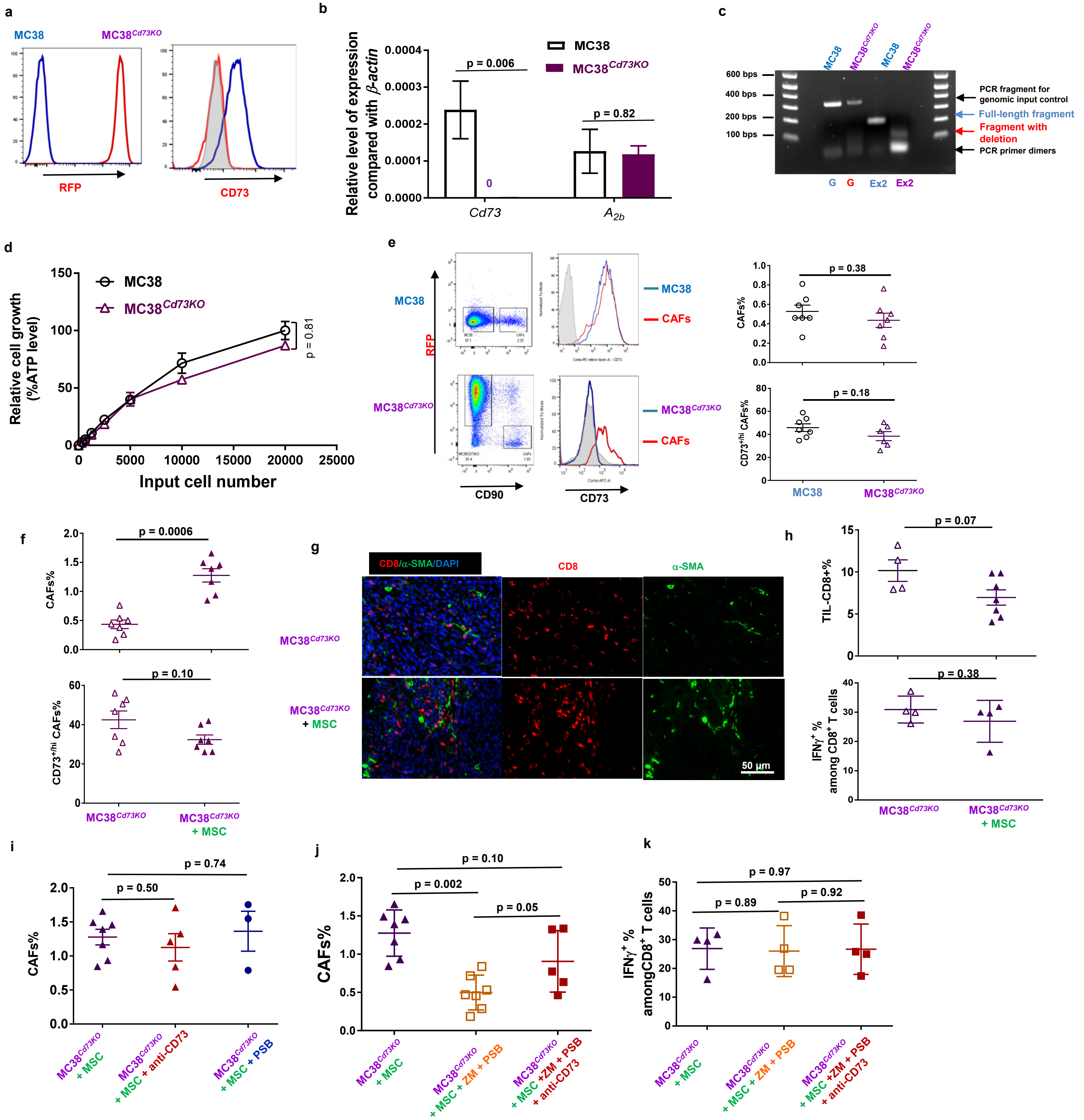

Figure S8. MC38^{Cd73KO} tumors engineered via CRISPR/Cas9-based knockout system in the presence of adequate fibroblastic stroma recapitulate the collaborative therapeutic effects of adenosinergic antagonism and CD73-neutralization. (a) *Cd73*-CRISPR/Cas9 KO plasmids (Santa Cruz, sc-423919) and *Cd73*-HDR plasmids (Santa Cruz, sc-423919-HDR) were co-transfected into MC38 tumors using Invitrogen Lipofectamine LTX transfection reagent. Forty-eight hours later, the transfected MC38 cells were first subjected to a 5-day puromycin selection followed by single cell cloning via FACSort of RFP^{high} cells. Representative FACS plots of an established MC38^{Cd73KO} clone showing its high level of RFP expression with accompanied lost of CD73 expression compared with those of MC38 control. (b) Real-time RT-PCR analysis and confirmation of *Cd73KO* in the established MC38^{Cd73KO} clone compared with those in MC38 tumors. (c) Genomic DNA PCR-based conformation of a deletion within the targeted *Cd73* Exon 2 sequences (Ex2) in the established MC38^{Cd73KO} clone but not MC38 cells. G is for amplification of a genomic DNA fragment serving as input control. (d) Comparative analysis of cellular growth rate between the MC38^{Cd73KO} clone and MC38 cells cultured for 48 H using the ATPLite kit. (e) Comparative analysis of CAF abundancy and the level of CD73 expression within the TME of the MC38^{Cd73KO} clone and MC38 tumors. were inoculated s.c. into the WT mice. (f) Comparative analysis of the percentage of total CAFs and CD73$^{hi/+}$ CAFs in the TME of MC38^{Cd73KO} without or with MSC co-inoculation. (g) Representative IHC analysis of CD8^{+} T cell and α-SMA^{+} cells distribution within the TME of MC38^{Cd73KO} tumors without or with MSC co-inoculation. (h) The percentage of TIL-CD8 T cells and IFN-γ producing CTLs in the TME of MC38^{Cd73KO} without or with MSC co-inoculation were analyzed via FACS. (I - k) The percentage of total CAFs (I, j) and IFN-γ producing CTLs (k) in the TME of MC38^{Cd73KO} tumors with MSC co-inoculation following various treatment was analyzed via FACS. Source data are provided in the Source Data file.
