# Supplementary material for: CD73 on cancer-associated fibroblasts enhanced by the A2B-mediated feedforward circuit enforces an immune checkpoint
Source: Nat Commun. 2020 Jan 24;11:515. doi: 10.1038/s41467-019-14060-x (PMC6981126; doi:10.1038/s41467-019-14060-x)
Supplement: Supplementary file 2 — Reporting Summary [file 41467_2019_14060_MOESM2_ESM.pdf]

nature research

Corresponding author(s): Yan Cui

Last updated by author(s): Dec 4, 2019

Reporting Summary

Nature Research wishes to improve the reproducibility of the work that we publish. This form provides structure for consistency and transparency in reporting. For further information on Nature Research policies, see Authors & Referees and the Editorial Policy Checklist .

Statistics

For all statistical analyses, confirm that the following items are present in the figure legend, table legend, main text, or Methods section.

n/a	Confirmed	
☐	☒	The exact sample size (*n*) for each experimental group/condition, given as a discrete number and unit of measurement
☐	☒	A statement on whether measurements were taken from distinct samples or whether the same sample was measured repeatedly
☐	☒	The statistical test(s) used AND whether they are one- or two-sided<br>*Only common tests should be described solely by name; describe more complex techniques in the Methods section.*
☒	☐	A description of all covariates tested
☐	☒	A description of any assumptions or corrections, such as tests of normality and adjustment for multiple comparisons
☒	☐	A full description of the statistical parameters including central tendency (e.g. means) or other basic estimates (e.g. regression coefficient) AND variation (e.g. standard deviation) or associated estimates of uncertainty (e.g. confidence intervals)
☒	☐	For null hypothesis testing, the test statistic (e.g. *F*, *t*, *r*) with confidence intervals, effect sizes, degrees of freedom and *P* value noted<br>*Give P values as exact values whenever suitable.*
☒	☐	For Bayesian analysis, information on the choice of priors and Markov chain Monte Carlo settings
☒	☐	For hierarchical and complex designs, identification of the appropriate level for tests and full reporting of outcomes
☒	☐	Estimates of effect sizes (e.g. Cohen's *d*, Pearson's *r*), indicating how they were calculated

Our web collection on statistics for biologists contains articles on many of the points above.

Software and code

Policy information about availability of computer code

Data collection	BD FACSDiva software V8.0, BioRad CFX384, PerkinElmer Vectra3
Data analysis	FlowJo V10, Prism 7.0 software (GraphPad), R2: Genomics Analysis and Visualization Platform (http://r2.amc.nl),<br>WEB-based GEne SeT AnaLysis Toolkit (http://www.webgestalt.org/option.php),<br>cuffdiff 2.0.2, Tophat 2.0.11, Shimadzu LC solution software, Adobe Photoshop CS, PerkinElmer Inform

For manuscripts utilizing custom algorithms or software that are central to the research but not yet described in published literature, software must be made available to editors/reviewers. We strongly encourage code deposition in a community repository (e.g. GitHub). See the Nature Research guidelines for submitting code & software for further information.

Data

Policy information about availability of data

All manuscripts must include a data availability statement. This statement should provide the following information, where applicable:

- Accession codes, unique identifiers, or web links for publicly available datasets
- A list of figures that have associated raw data
- A description of any restrictions on data availability

Source Data files for Figures and supplementary Figures are included in the submission. The RNA-seq data are available at Genes Expression Omnibus (GEO) under the number GSE141199 (https://www.ncbi.nlm.nih.gov/geo/query/acc.cgi?acc=GSE141199).

Field-specific reporting

Please select the one below that is the best fit for your research. If you are not sure, read the appropriate sections before making your selection.

☒ Life sciences ☐ Behavioural & social sciences ☐ Ecological, evolutionary & environmental sciences

For a reference copy of the document with all sections, see nature.com/documents/nr-reporting-summary-flat.pdf

Life sciences study design

All studies must disclose on these points even when the disclosure is negative.

Sample size	Experimental group sizes were designed to have n>=5 per group. To determine the effects of therapeutic treatment on tumor sizes, n>=7 per group was used based on our previous results of murine tumor (EG7) treatment/progression experiments. The coefficient of variation for tumor volume was usually ≤ 0.4. The group size of 7 will provide 90% power to detect a two-fold difference at any given time between two groups with a significance, a set at 0.05.
Data exclusions	No exclusions. All experimental results were included.
Replication	All experiments were repeated and results were reproducible.
Randomization	All the tumor bearing mice were sequentially assigned based on their ear tag number into various control and treatment groups.
Blinding	The investigators were not blinded to the experiment design and treatments.

Reporting for specific materials, systems and methods

We require information from authors about some types of materials, experimental systems and methods used in many studies. Here, indicate whether each material, system or method listed is relevant to your study. If you are not sure if a list item applies to your research, read the appropriate section before selecting a response.

Materials & experimental systems

n/a	Involved in the study	
☐	☒	Antibodies
☐	☒	Eukaryotic cell lines
☒	☐	Palaeontology
☐	☒	Animals and other organisms
☒	☐	Human research participants
☒	☐	Clinical data

Methods

n/a	Involved in the study	
☒	☐	ChIP-seq
☐	☒	Flow cytometry
☒	☐	MRI-based neuroimaging

Antibodies

Antibodies used	anti-CD4 (RM4-5), anti-CD8α (53-6.7), anti-CD11b (M1/70), anti-CD19 (HIB19), anti-CD25 (PC61), anti-CD31 (MEC13.3), anti-CD45.2 (104), anti-CD69 (H1.2F3), anti-CD90.2 (53-2.1), anti-NK1.1 (PK136) and anti-Annexin, all from BD Biosciences (San Jose, CA); anti-CD73 (TY/11.8), anti-IFN-γ (XMG1.2), anti-pZAP-70/SYK (n3kobu5) and 7-AAD (7-Aminoactinomycin D) all from eBioscience (San Diego, CA); anti-mouse gp38 (8.1.1) was from Biolegend (San Diego, CA). Anti-HIF-1 (D2U3T) was from Cell signaling (Boston, MA); Anti-ER-TR7 was from Abcam (Cambridge, MA); Alexa Fluor – conjugated goat secondary Abs were from Life Technologies (Carlsbad, CA). Functional-grade purified anti-CD3ε (17A2) was from Biolegend. Anti-CD73 (clone TY/23) and rat IgG2a isotype control (clone 2A3) for in vivo study were obtained from Bio X Cell (West Lebanon, NH).
Validation	All the antibodies were first confirmed with their specific staining using known positive cells and negative cells with expected pattern.

Eukaryotic cell lines

Policy information about cell lines

Cell line source(s)	Ova-expressing E.G7-OVA (EG7) cells were purchased from ATCC (Manassas, VA) and maintained in DMEM (Invitrogen, Carlsbad, CA) supplemented with 10% FBS and 0.4 mg/ml G418. MC38 cells were obtained from Kerafast (Boston, MA).
Authentication	No additional authentication was performed.
Mycoplasma contamination	Periodically tested with the culture medium via the standard PCR based assay.

Commonly misidentified lines (See ICLAC register) | *Name any commonly misidentified cell lines used in the study and provide a rationale for their use.*

Animals and other organisms

Policy information about studies involving animals; ARRIVE guidelines recommended for reporting animal research

Laboratory animals	C57BL/6 mice were purchased from Charles River (Wilmington, MA). CD73null (B6.129S1-Nt5etm1Lft/J), GFP-Tg (C57BL/6-Tg (UBC-GFP)30Scha/J), and A2Bnull (B6.129P2-Adora2btm1Till/J) mice were obtained from the Jackson Laboratory (Bar Harbor, ME).
Wild animals	None.
Field-collected samples	None.
Ethics oversight	All experimental protocols involving experimental animals were approved by the Augusta University Institutional Animal Care and Use Committee. De-identified frozen human colorectal cancer specimens were obtained from Georgia Cancer Center Biorepository following protocols approved by the Augusta University Institutional Review Board committees. No population data were obtained.

Note that full information on the approval of the study protocol must also be provided in the manuscript.

Flow Cytometry

Plots

Confirm that:

- [x] The axis labels state the marker and fluorochrome used (e.g. CD4-FITC).
- [x] The axis scales are clearly visible. Include numbers along axes only for bottom left plot of group (a 'group' is an analysis of identical markers).
- [ ] All plots are contour plots with outliers or pseudocolor plots.
- [x] A numerical value for number of cells or percentage (with statistics) is provided.

Methodology

Sample preparation	As described in detail in the method section, under the "CAF isolation, culture and purification" , the EG7 tumor tissues from mice were dissected and minced into small pieces in DMEM supplemented with 10% fetal bovine serum (FBS) and 1% penicillin-streptomycin (Life Technologies). They were digested with 0.8 mg/ml Dispase, 0.2 mg/ml Collagenase P, and 0.1 mg/ml DNase I (all from Roche, Basel, Switzerland) at 37°C with shaking for 60 min. At 30 min intervals, digested tissues were pipetted to facility dissociation and dispersed single cell were collected and kept on ice. For purifying fresh CAFs, the aforementioned digested EG7 tumor suspension was filtered through a 70 um strainer and cells were stained with CD45.2 (104), CD31 (MEC13.3) and GP38 (8.1.1) to obtain CD45- CD31- GP38+ CAFs via low pressure FACsorting with FACS Aria using 100 um nozzle (BD Biosciences). Cells were suspended in FACS buffer for flow assay.
Instrument	4-Laser LSRII with HTS flow cytometer
Software	BD FACSDiva software V8.0 and FlowJo V10.
Cell population abundance	Expressed as the percentage of total populations.
Gating strategy	The cellular population stained with isotype control antibody or unstained negative control were used for determining the division point for negative and positive signals and gate boundary.

- [x] Tick this box to confirm that a figure exemplifying the gating strategy is provided in the Supplementary Information.
